# Supplementary material for: General synthesis and atomic arrangement identification of ordered Bi–Pd intermetallics with tunable electrocatalytic CO2 reduction selectivity
Source: Nat Commun. 2024 Feb 21;15:1573. doi: 10.1038/s41467-024-46072-7 (PMC10881518; doi:10.1038/s41467-024-46072-7)
Supplement: Supplementary file 1 — Supplementary Information [file 41467_2024_46072_MOESM1_ESM.pdf]

For submission to *Nature Communications*

Supplementary Information File for

General Synthesis and Atomic Arrangement Identification of Ordered Bi-Pd Intermetallics with Tunable Electrocatalytic CO_2 Reduction Selectivity

Wenjin Guo[1,2], Guangfang Li[3], Chengbo Bai[1], Qiong Liu[2], Fengxi Chen[1], Rong Chen [1,4*]

[1]State Key Laboratory of New Textile Materials & Advanced Processing Technologies, Wuhan Textile University, Wuhan, 430200

[2]School of Chemistry and Environmental Engineering, Wuhan Institute of Technology, Wuhan 430205, PR China

[3]Key Laboratory of Material Chemistry for Energy Conversion and Storage (Ministry of Education), Hubei Key Laboratory of Material Chemistry and Service Failure, Huazhong University of Science and Technology, Wuhan 430074, PR China

[4]Henan Institute of Advanced Technology, Zhengzhou University, Zhengzhou 450002, PR China

*Corresponding author: Prof. R. Chen, E-mail address: rchenhku@hotmail.com

Table of Contents

**Section 1**	Characterization	Page 3
**Section 2**	The exploration of formation mechanism	Fig.1-6
**Section 3**	Characterization and analysis of ordered structures	Fig.7
**Section 4**	X-ray absorption spectroscopy analysis of Bi–Pd IMCs	Fig.8-9
**Section 5**	Elemental analysis and characterization	Fig.10-15
**Section 6**	CO_2 electroreduction performance	Fig.16-17
**Section 7**	CO_2 electroreduction stabilities	Fig.18-19
**Section 8**	The exploration of size effect	Fig.20-22
**Section 9**	DFT calculations	Fig.23-30
**Section 10**	Potential-resolved in-situ FT-IR full-spectra of Bi-Pd IMCs	Fig.31
**Section 11**	EXAFS fitting parameters	Table 1-2
**Section 12**	The reaction paths	Table 3-5

Characterization

The element content and crystal phase of the obtained samples were characterized by powder X-ray diffraction (PXRD, Bruker axs D8, Germany) with discover Cu Kα=1.5406 Å, and flame atomic absorption spectrometer (AAS, SP 3520AA, China). The morphology and surface atomic arrangement of the obtained samples were characterized by scanning electron microscope (SEM, ZEISS Gemini SEM 300, Germany) operating at 10.0 kV and double Cs-correctors transmission electron microscopy (Double CS-corrected TEM, FEI-Titan Cubed Themis G2 60-300, USA) operating at 300 kV. The element content and valence state were analyzed by an X-ray photoelectron spectrometer (XPS, VG Multilab 2000 VG Inc, USA), and the binding energy was charge-corrected with reference to the C 1s peak at 284.8 eV. Raman spectra were recorded on a Laser Microscopic Confocal Raman Spectrometer (DXR3, USA) and collected in the range of 100 to 3000 cm^{-1} using a 532 nm laser. The in-situ Fourier-transform infrared spectroscopy (FTIR, Thermo Nicolet iS50, USA) were applied to monitor the generation of intermediates and products in CO_2RR with the increased potentials.

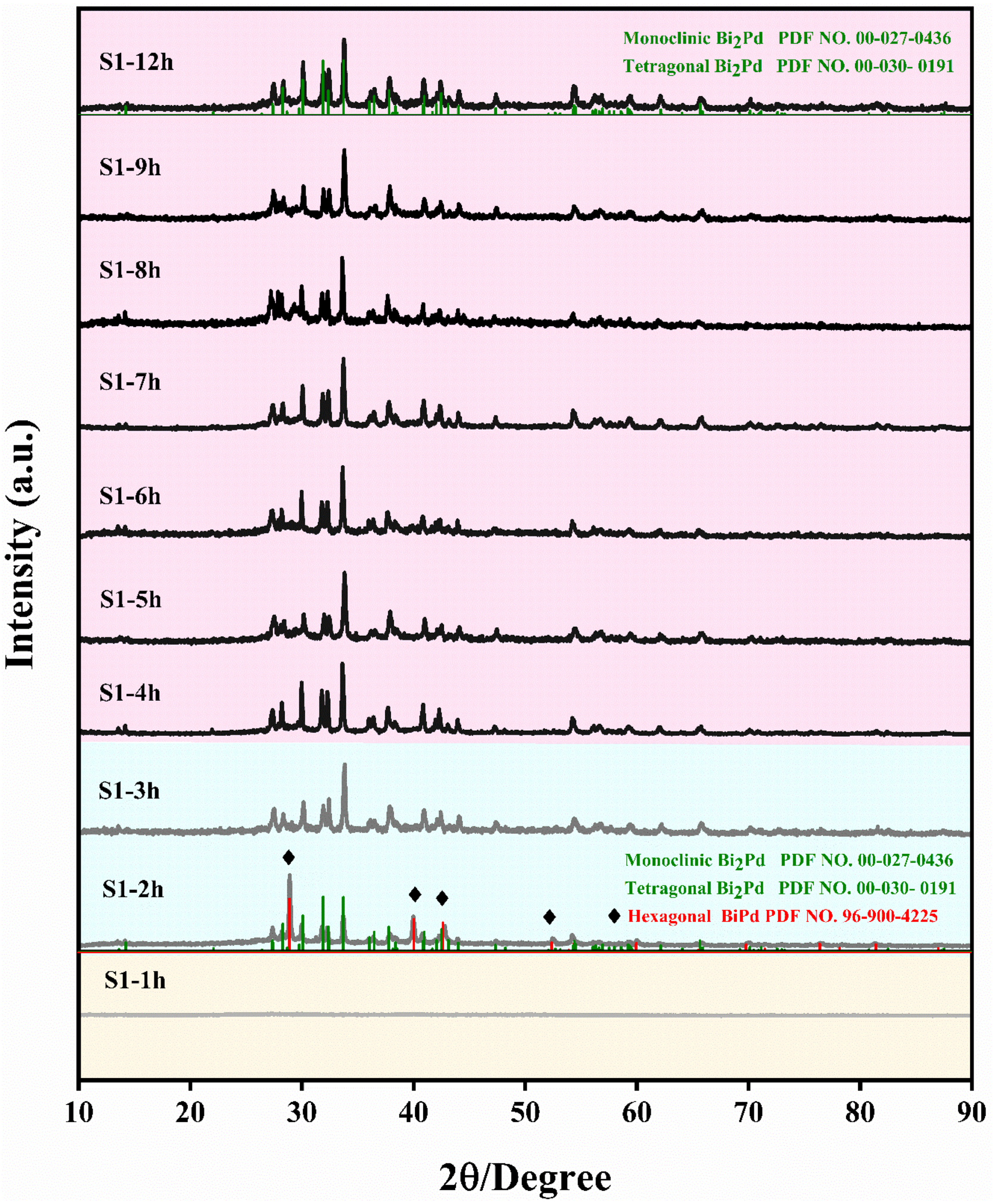

Supplementary Fig.1| The exploration of formation mechanism. XRD patterns of the S1-Bi_2Pd products obtained at various reaction times.

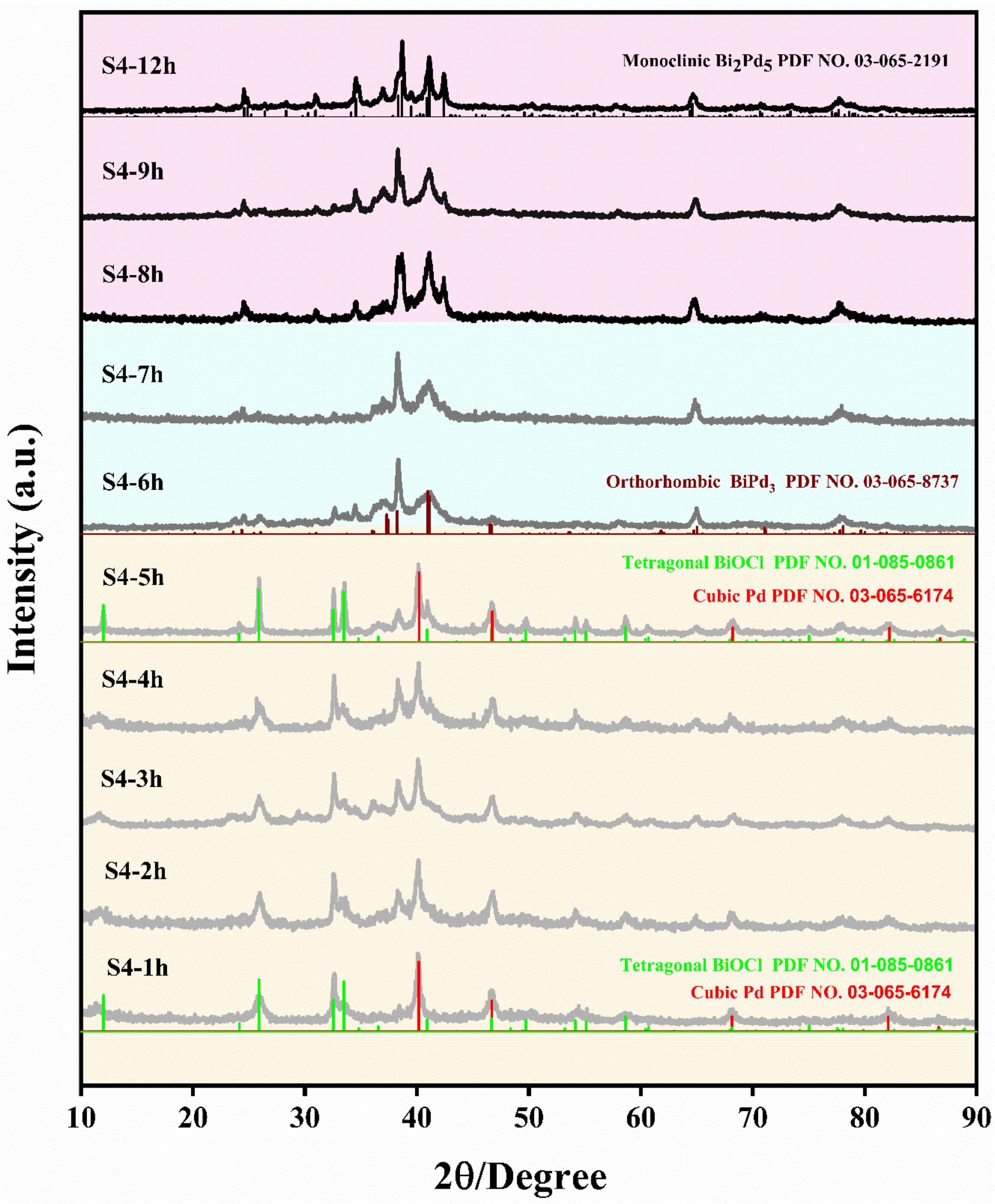

Supplementary Fig.2| The exploration of formation mechanism. XRD patterns of the S4-Bi_2Pd_5 products obtained at various reaction times.

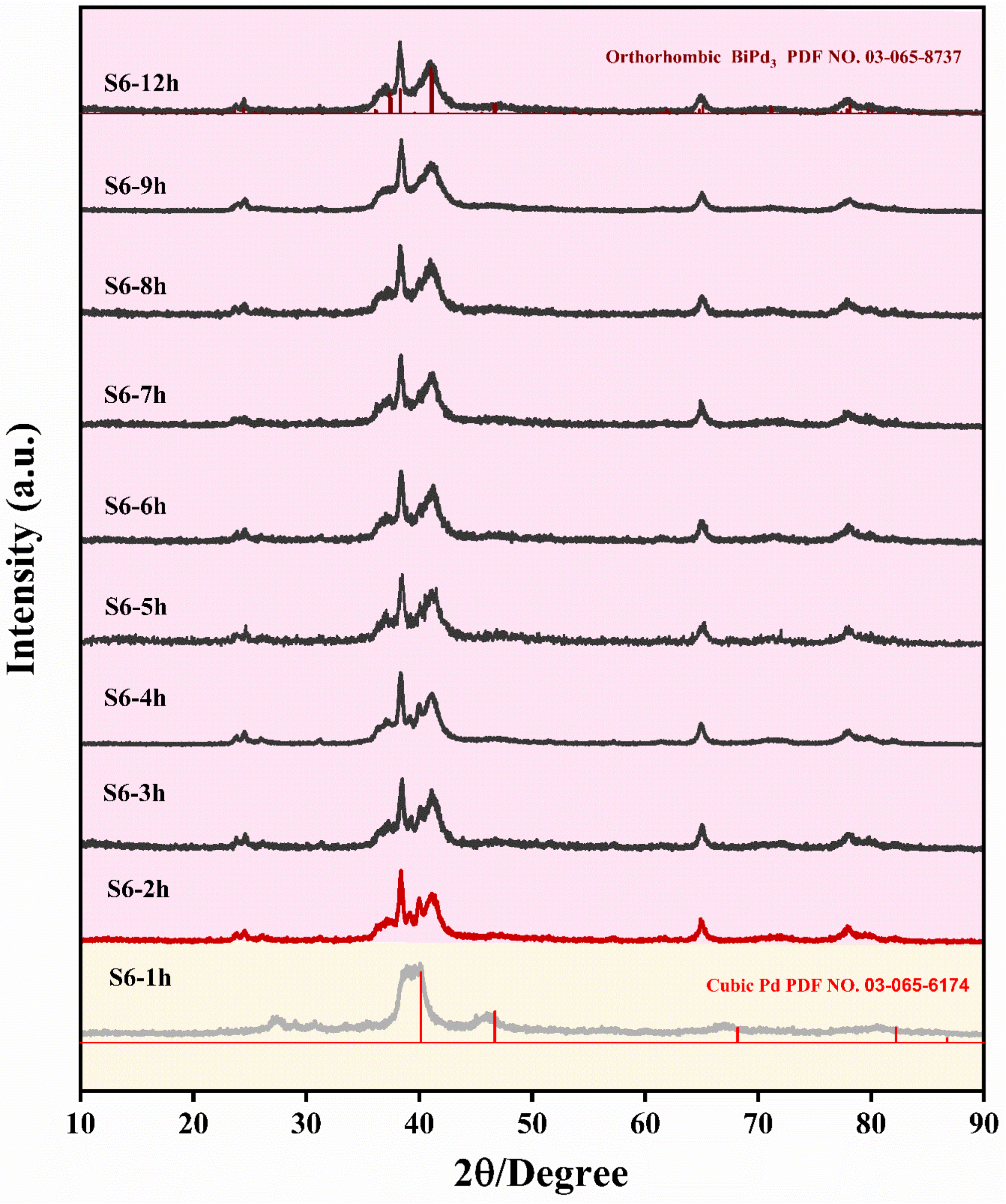

Supplementary Fig.3| The exploration of formation mechanism. XRD patterns of the S6-$BiPd_3$ products obtained at various reaction times.

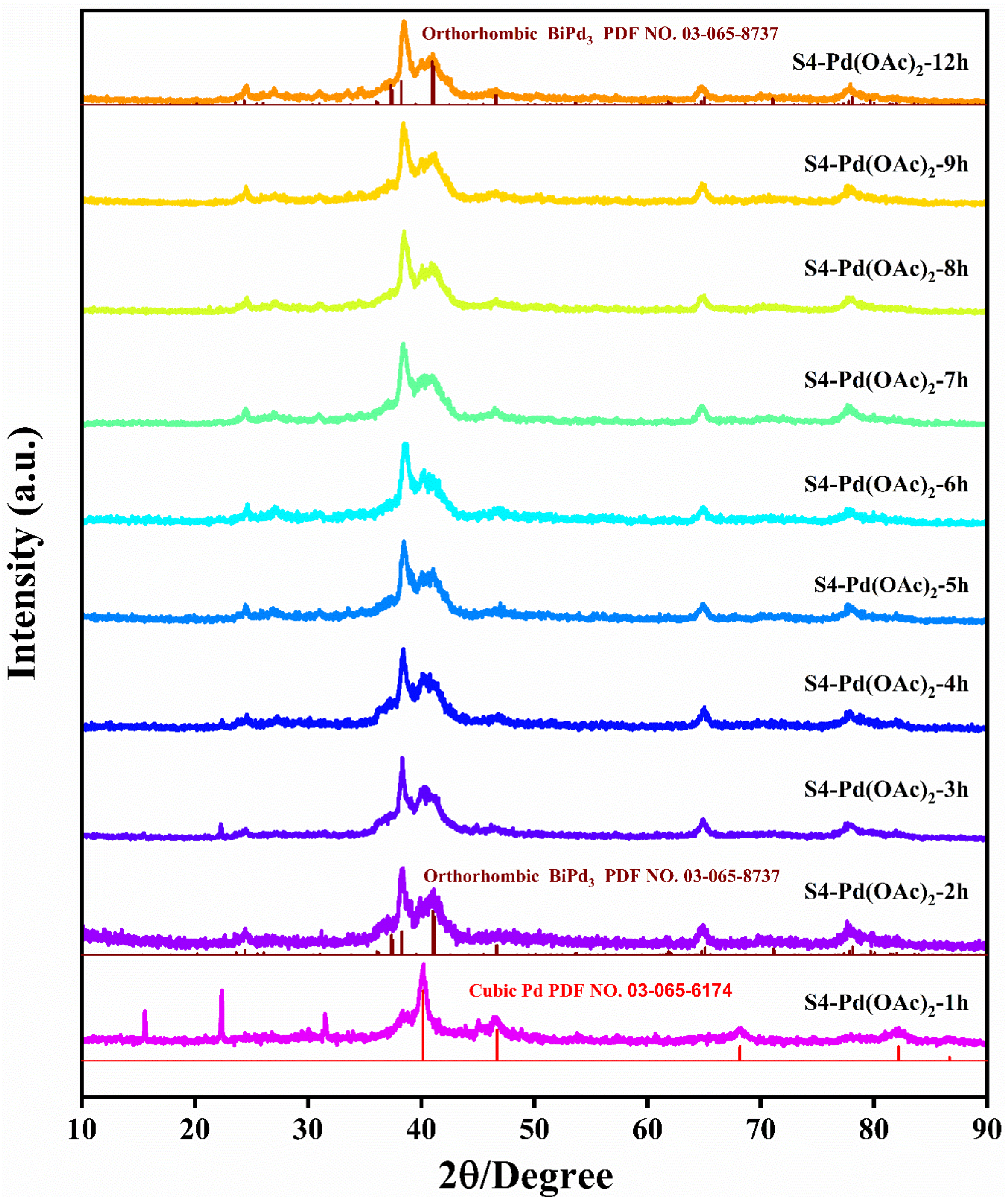

Supplementary Fig.4| The exploration of formation mechanism. XRD patterns of the S4-Bi_2Pd_5 products using $Pd(OAc)_2$ ($Pd(acetate)_2$) as the Pd precursor obtained at various reaction times.

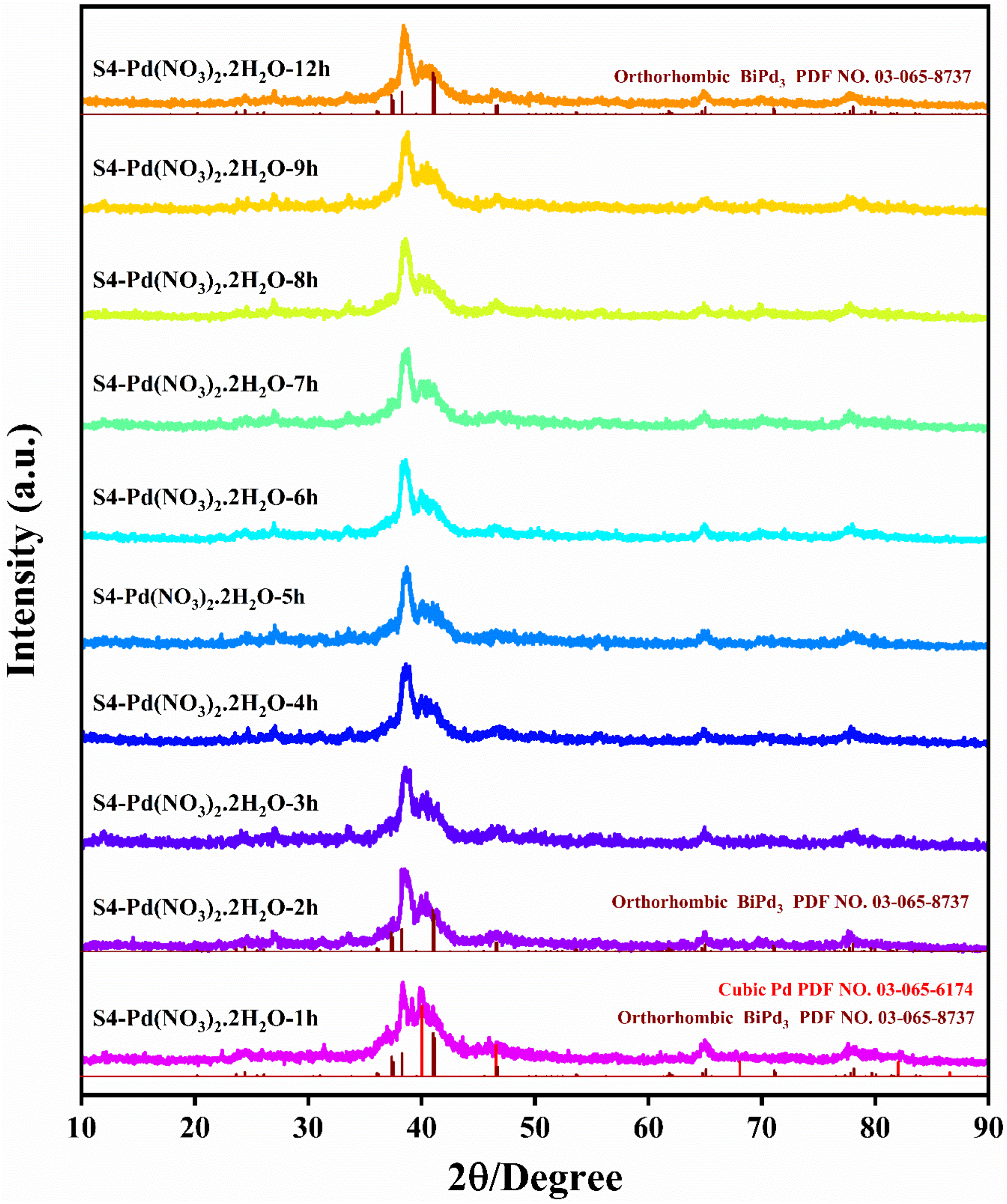

Supplementary Fig.5| The exploration of formation mechanism. XRD patterns of the S4-Bi_2Pd_5 products using $Pd(NO_3)_2.2H_2O$ as the Pd precursor obtained at various reaction times.

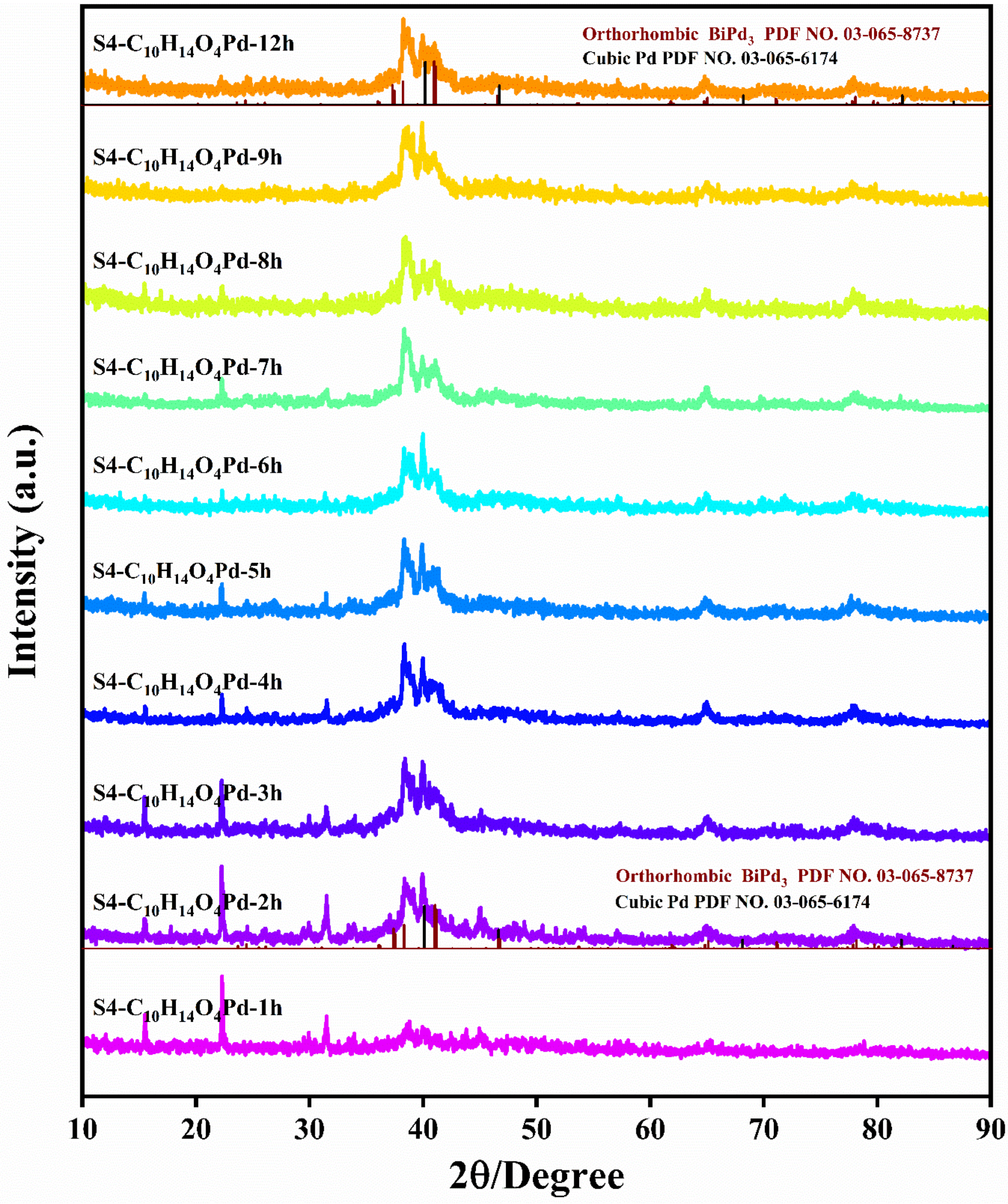

Supplementary Fig.6| The exploration of formation mechanism. XRD patterns of the S4-Bi_2Pd_5 products using $C_{10}H_{14}O_4Pd$ ($Pd(acac)_2$) as the Pd precursor obtained at various reaction times.

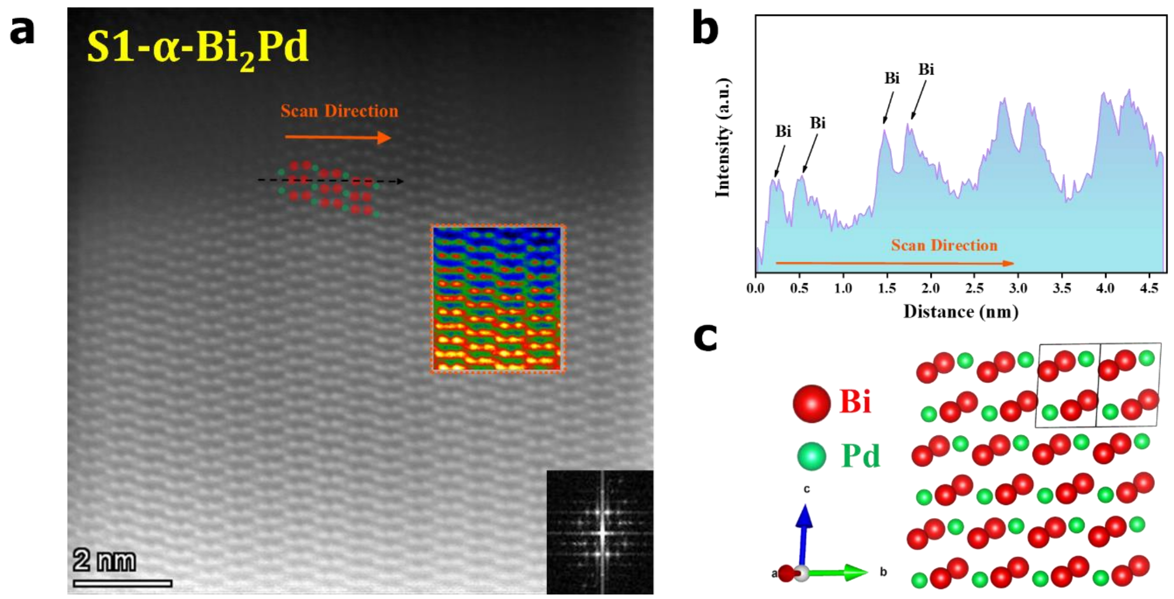

Supplementary Fig.7| Characterization and analysis of ordered structures. a Aberration-corrected HAADF–STEM images of α-Bi_2Pd. Inset: corresponding FFT pattern, bright contrast file in the orange box and diagram of atomic arrangement and spacing. **b** Intensity profiles measured from HAADF–STEM images and **c** corresponding crystal structure (red and green spheres represent Bi and Pd atoms).

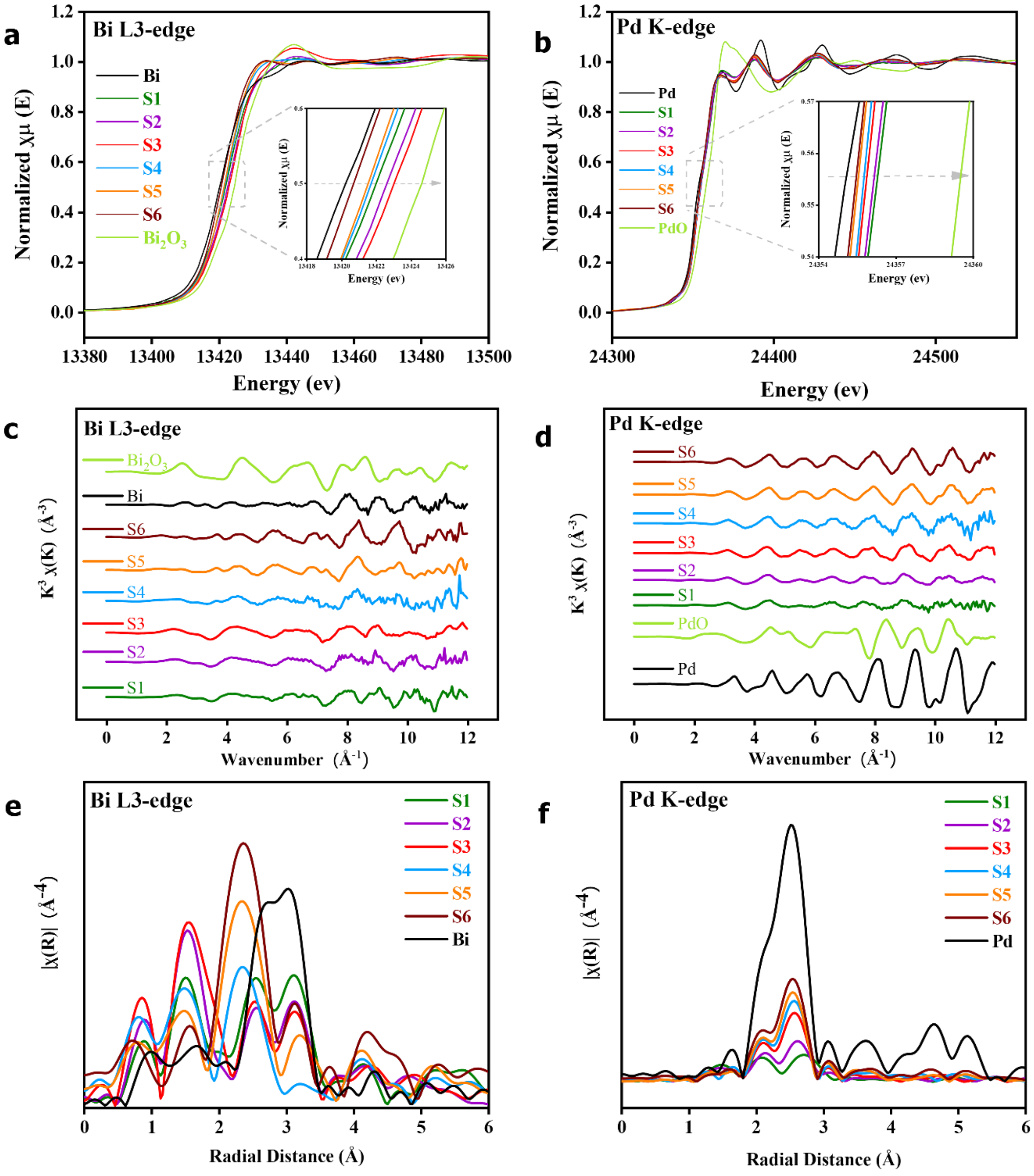

Supplementary Fig.8| X-ray absorption spectroscopy analysis of Bi–Pd IMCs. a Normalized XANES at the Bi L3-edge and **b** Pd K-edge. **C-f** k^3 -weighted χ(k) and χ(R)function of the EXAFS spectra at the Bi L3-edge and Pd K-edge.

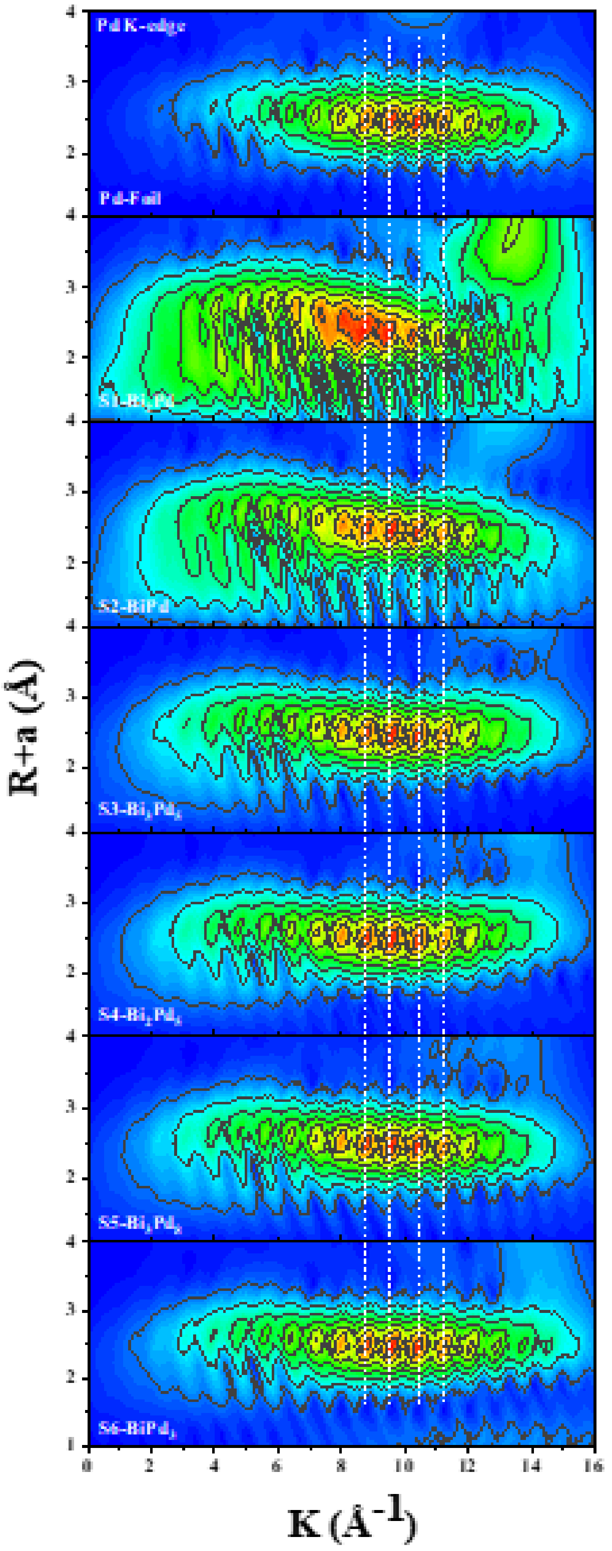

Supplementary Fig.9| X-ray absorption spectroscopy analysis of Bi–Pd IMCs.

WT–EXAFS Pd K-edge spectra for Bi-Pd IMCs and Pd foil reference.

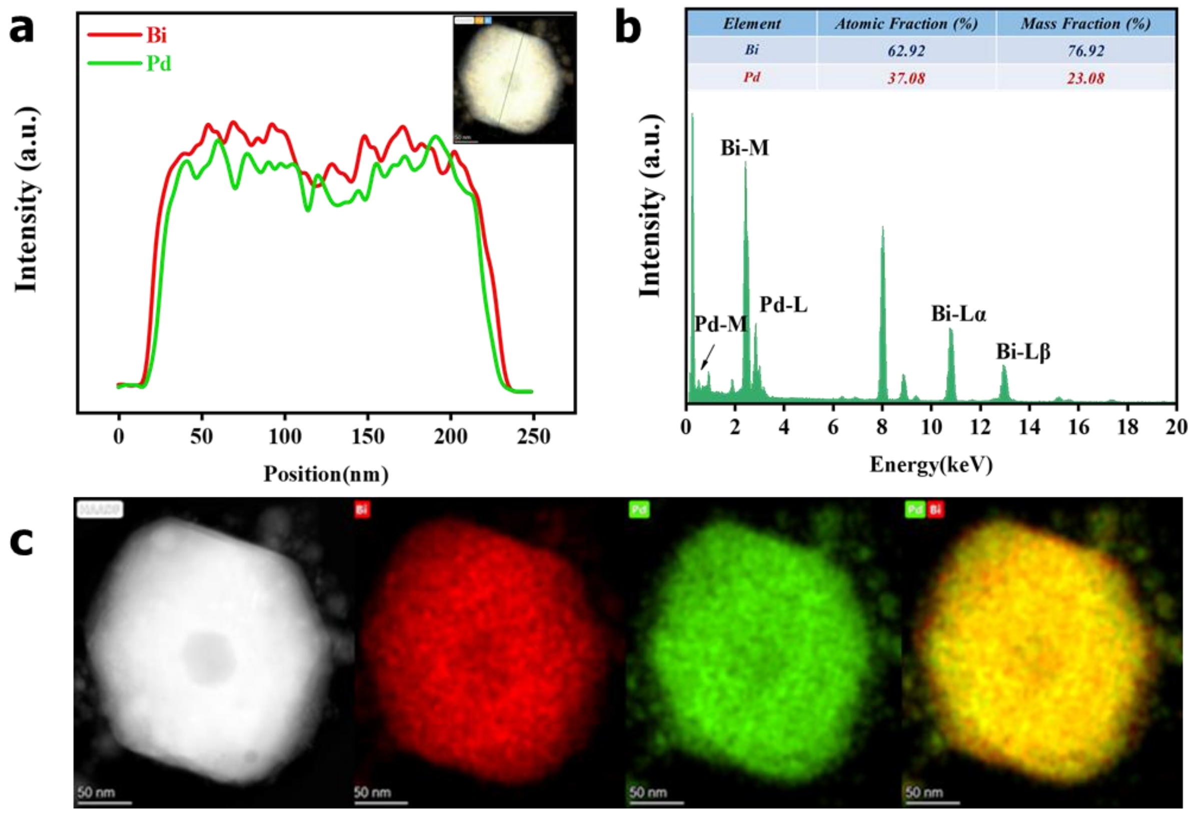

Supplementary Fig.10| Elemental analysis and characterization. a EDX line scan across S1-Bi_2Pd as indicated by the dashed line in the illustration **b** The STEM-EDX and corresponding mass and atomic fraction of the Pd and Bi elements in S1-Bi_2Pd. **c** STEM image and the corresponding elemental mappings of S1-Bi_2Pd.

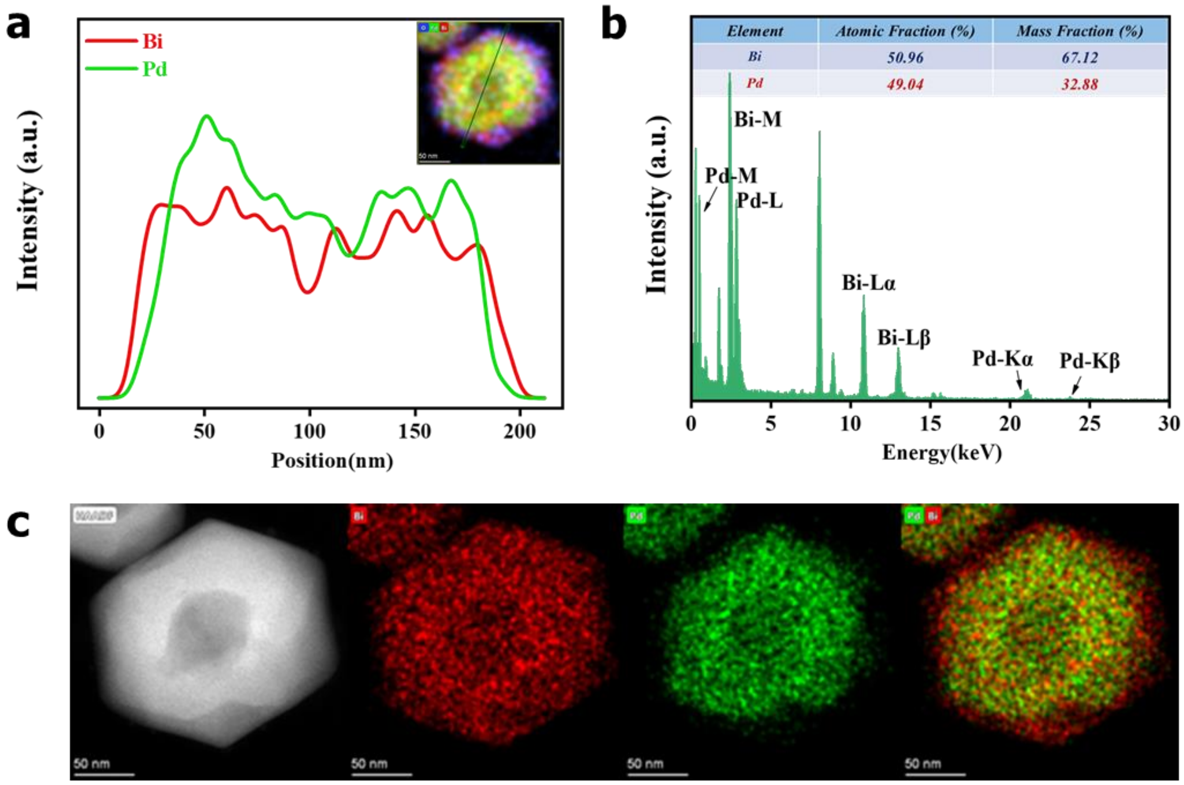

Supplementary Fig.11| Elemental analysis and characterization. a EDX line scan across S2-BiPd as indicated by the dashed line in the illustration **b** The STEM-EDX and corresponding mass and atomic fraction of the Pd and Bi elements in S2-BiPd. **c** STEM image and the corresponding elemental mappings of S2-BiPd.

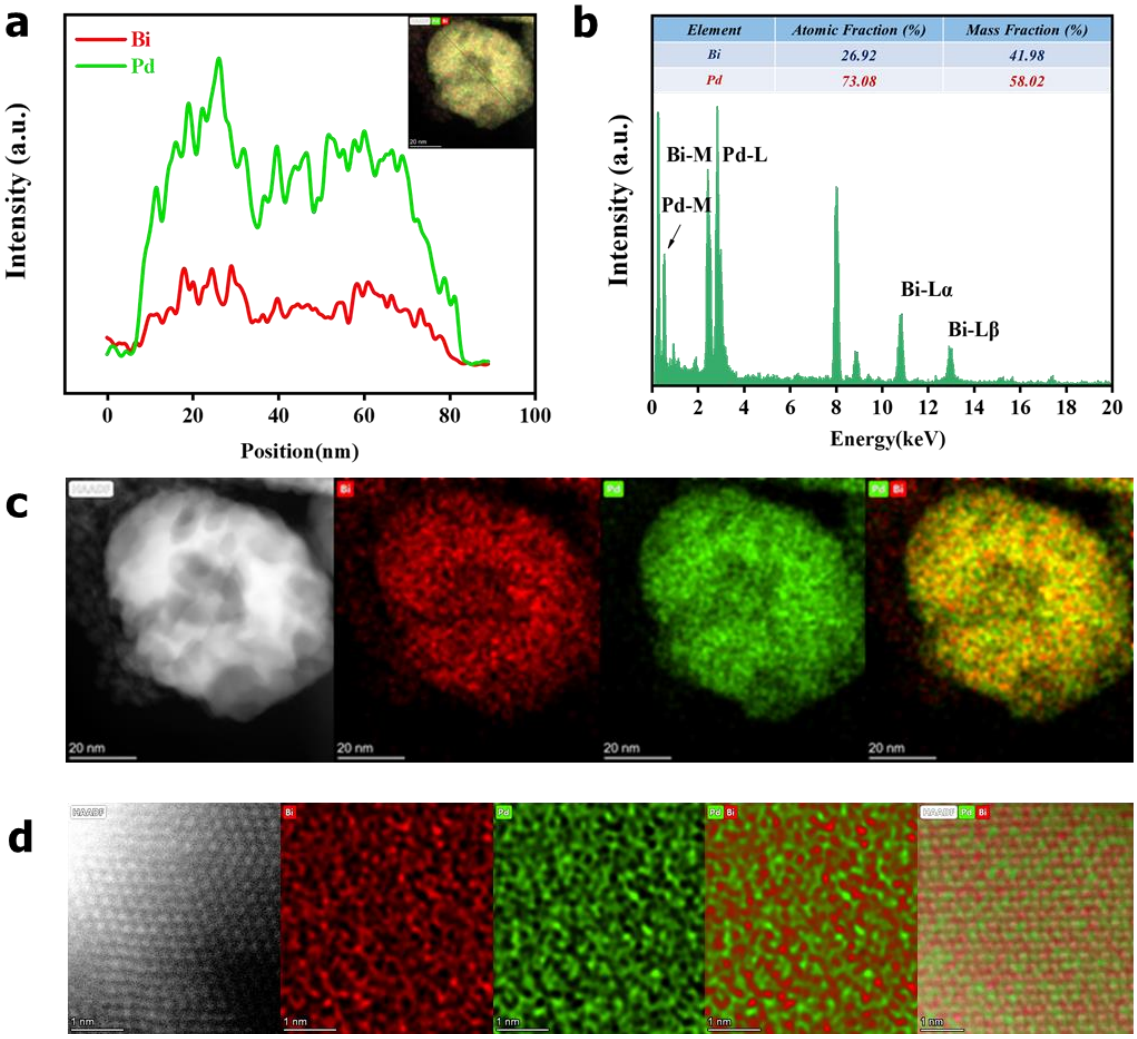

Supplementary Fig.12| Elemental analysis and characterization. a EDX line scan across S3-Bi_3Pd_5 as indicated by the dashed line in the illustration **b** The STEM-EDX and corresponding mass and atomic fraction of the Pd and Bi elements in S3-Bi_3Pd_5. **c** STEM image and the corresponding elemental mappings of S3-Bi_3Pd_5. **d** Atomic resolution EDX mapping of S3-Bi_3Pd_5.

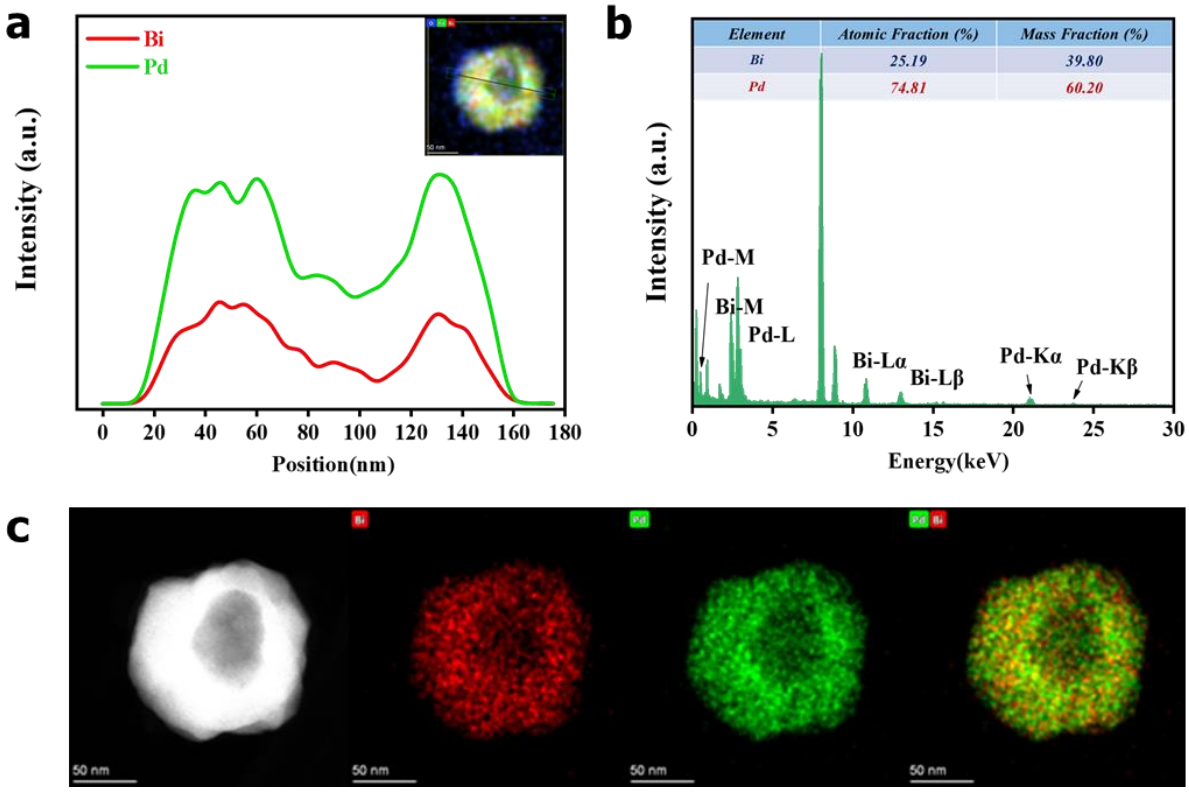

Supplementary Fig.13| Elemental analysis and characterization. a EDX line scan across S4-Bi_2Pd_5 as indicated by the dashed line in the illustration **b** The STEM-EDX and corresponding mass and atomic fraction of the Pd and Bi elements in S4-Bi_2Pd_5. **c** STEM image and the corresponding elemental mappings of S4-Bi_2Pd_5.

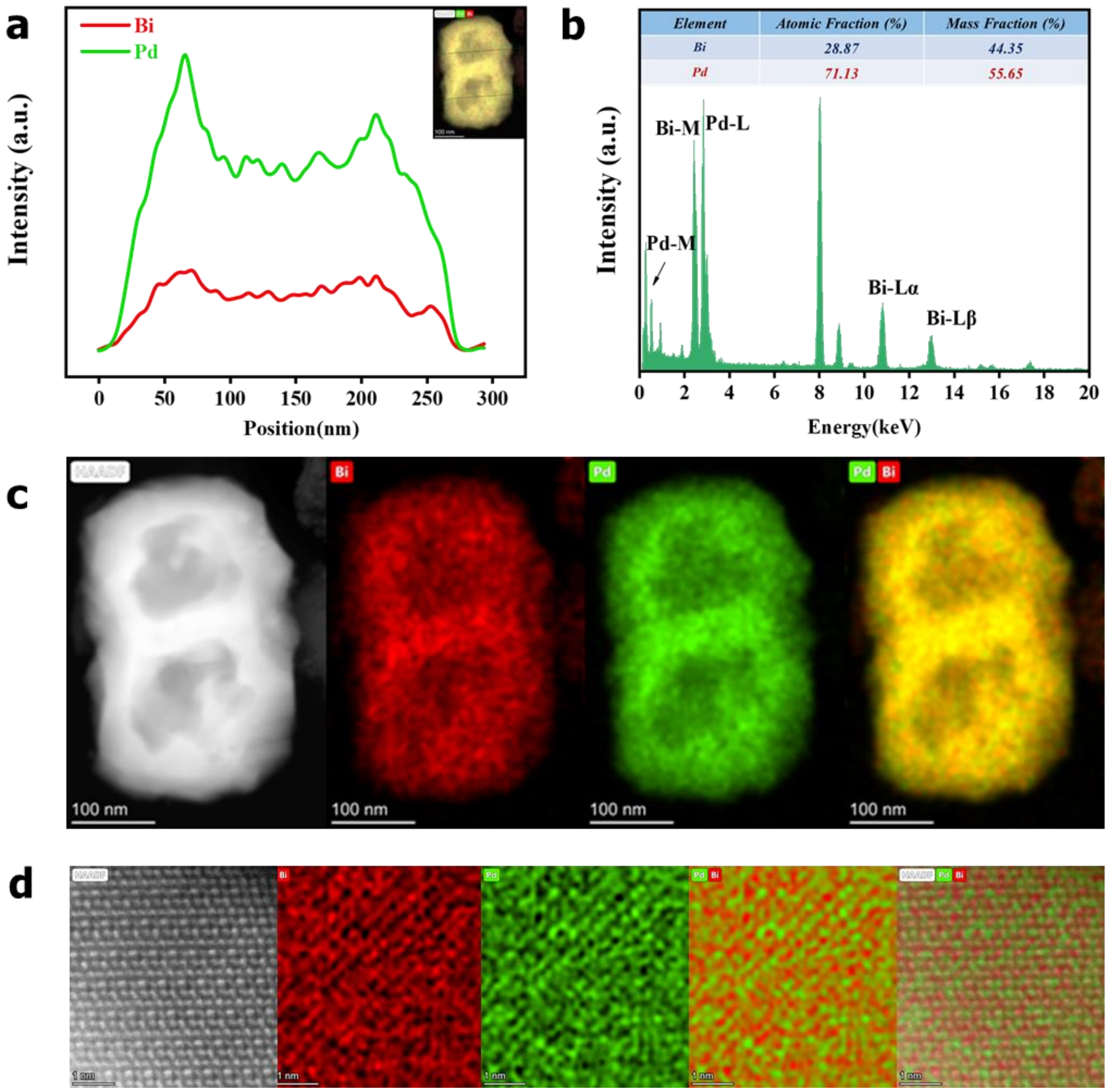

Supplementary Fig.14| Elemental analysis and characterization. a EDX line scan across S5-Bi_3Pd_8 as indicated by the dashed line in the illustration **b** The STEM-EDX and corresponding mass and atomic fraction of the Pd and Bi elements in S5-Bi_3Pd_8. **c** STEM image and the corresponding elemental mappings of S5-Bi_3Pd_8. **d** Atomic resolution EDX mapping of S5-Bi_3Pd_8.

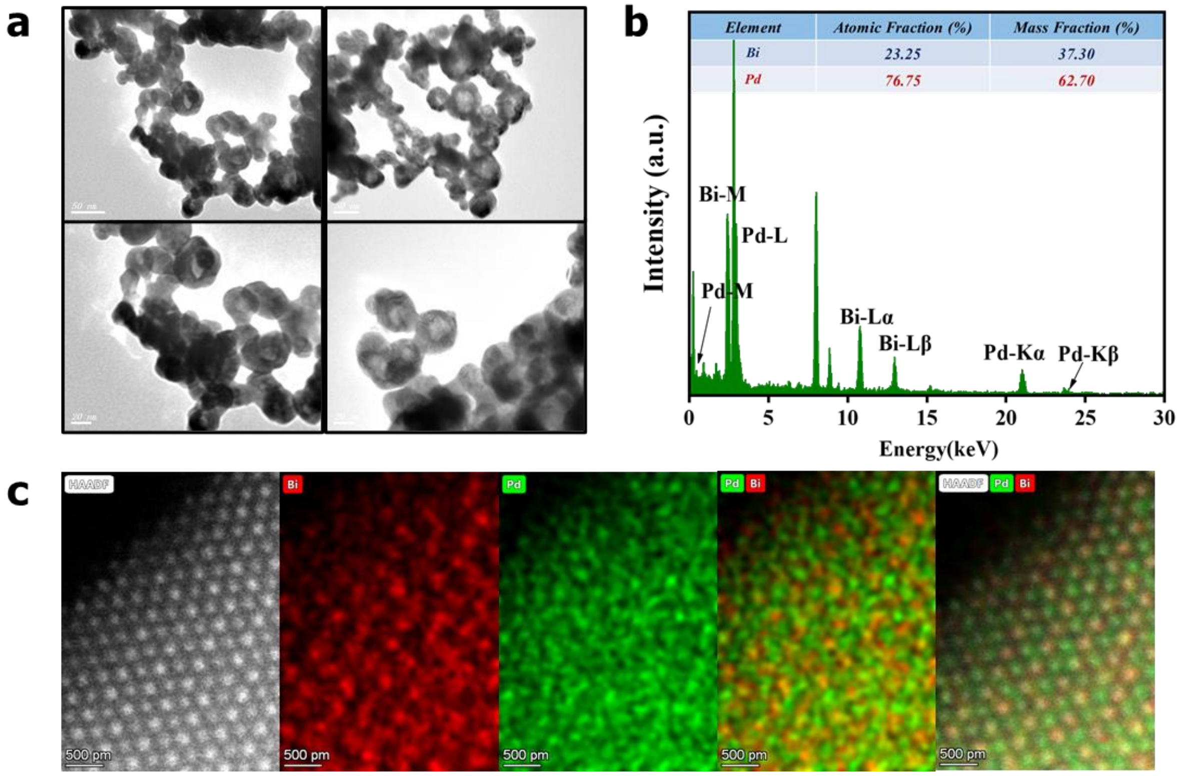

Supplementary Fig.15| Elemental analysis and characterization. a HRTEM image of S6-$BiPd_3$. **b** The STEM-EDX and corresponding mass and atomic fraction of the Pd and Bi elements in S6-$BiPd_3$. **c** Atomic resolution EDX mapping of S6-$BiPd_3$.

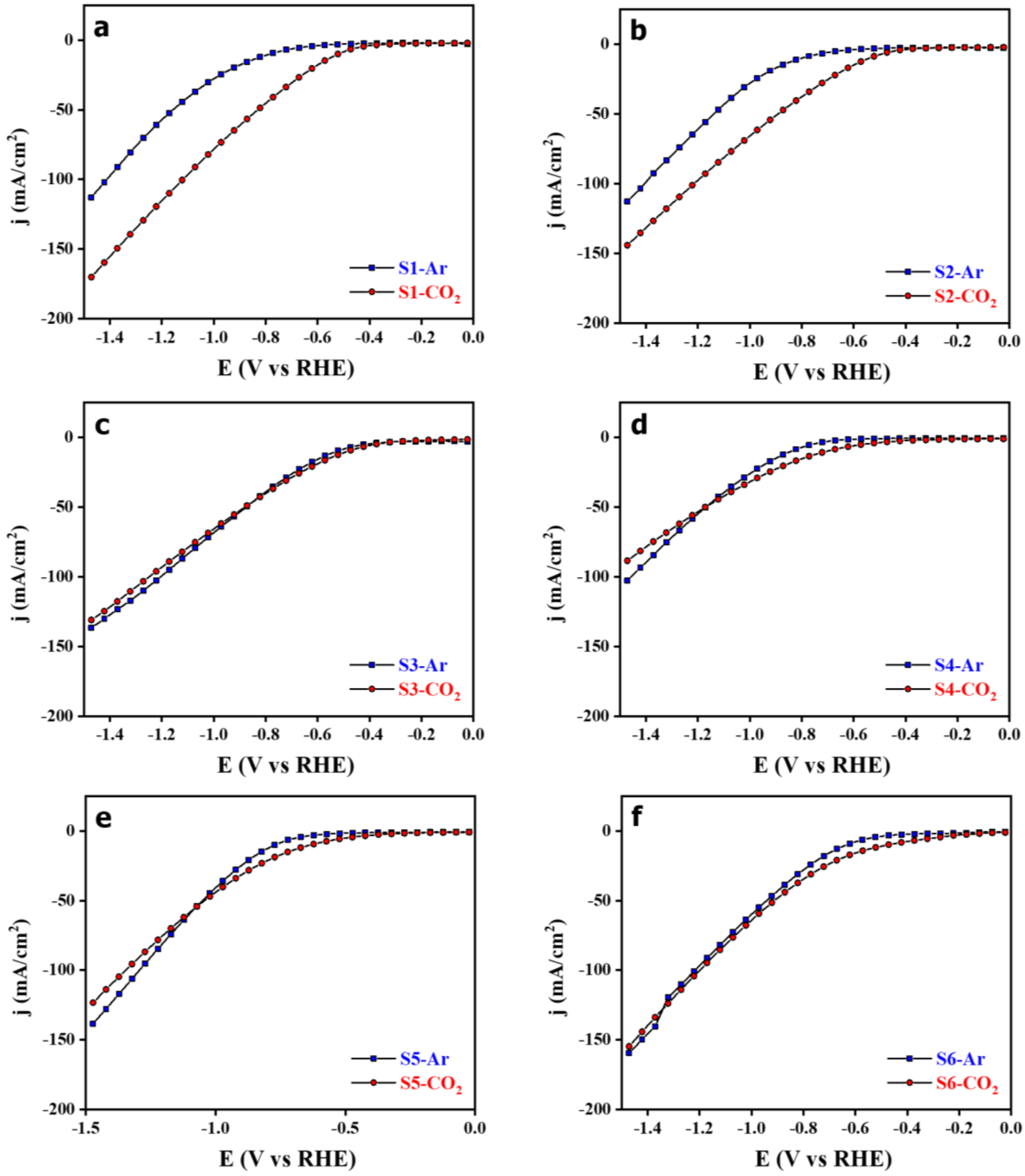

Supplementary Fig.16| CO_2 electroreduction performance. a-f LSV curves of the Bi-Pd IMCs in the Ar/CO_2.

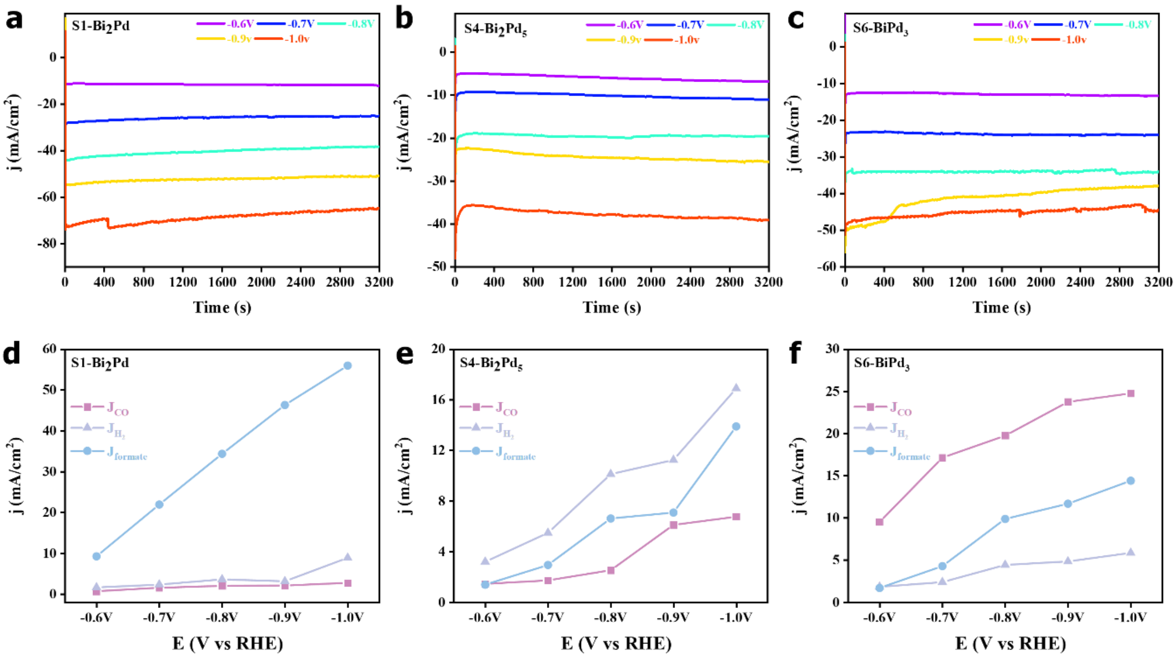

Supplementary Fig.17| CO_2 electroreduction performance. Constant potential electrolysis at each applied potential of **a** S1 **b** S4 **c** S6. Potential dependent partial current densities of **d** S1 **e** S4 **f** S6.

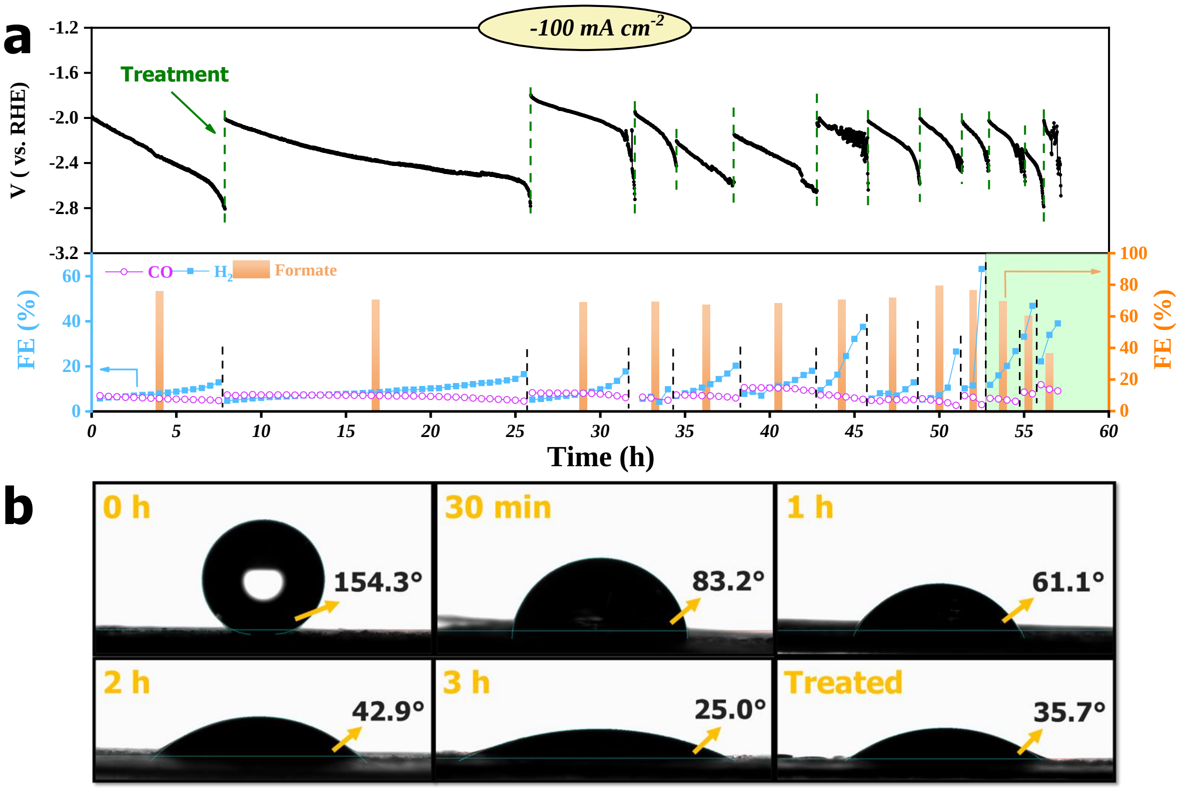

Supplementary Fig.18| CO_2 electroreduction stabilities. a Long-term stability test of S1 at -100 mA cm^{-2} in flow cell. **b** Contact angles of the cathode electrode surface at different reaction time and aftertreatment.

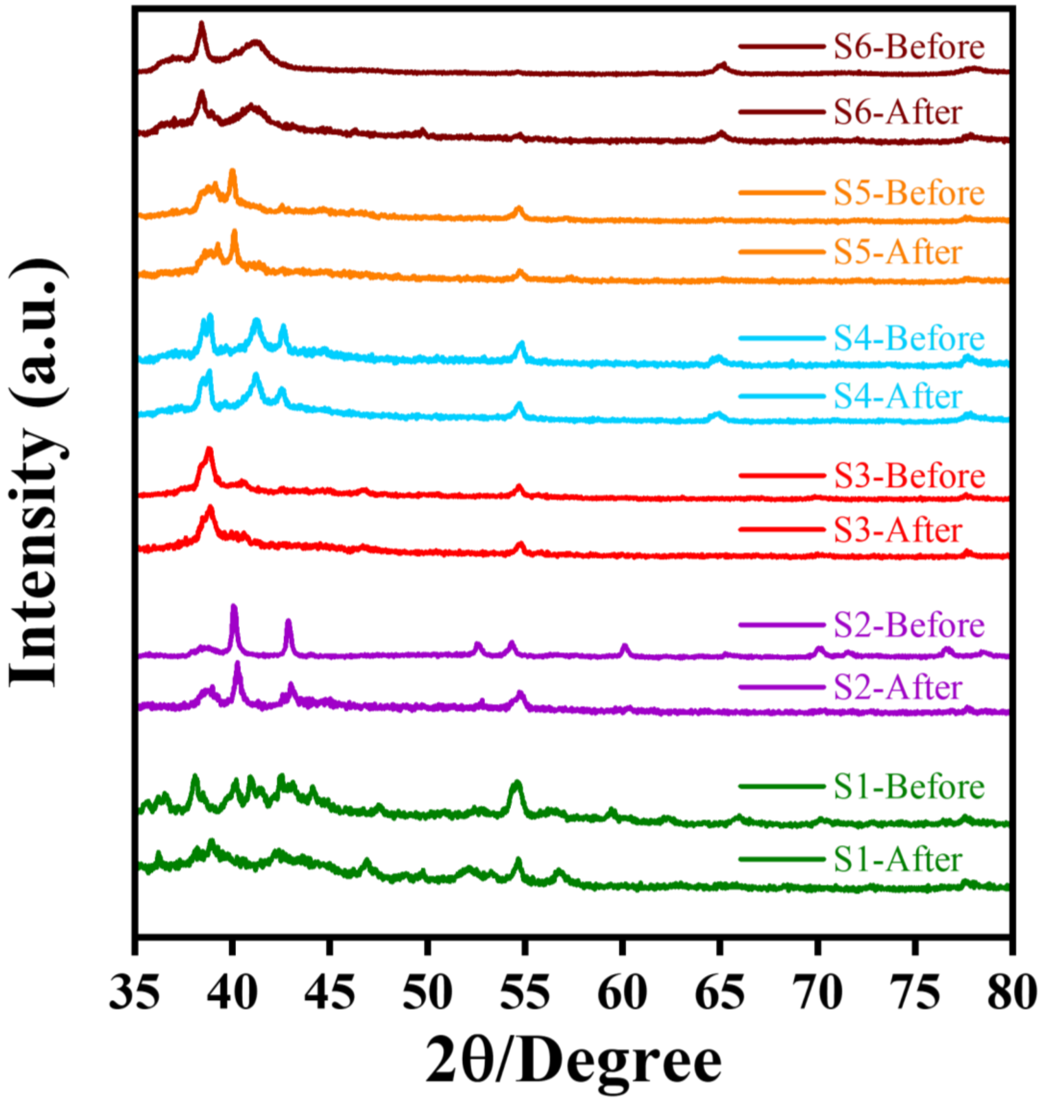

Supplementary Fig.19| CO_2 electroreduction stabilities. XRD patterns of the Bi-Pd IMCs before and after reaction.

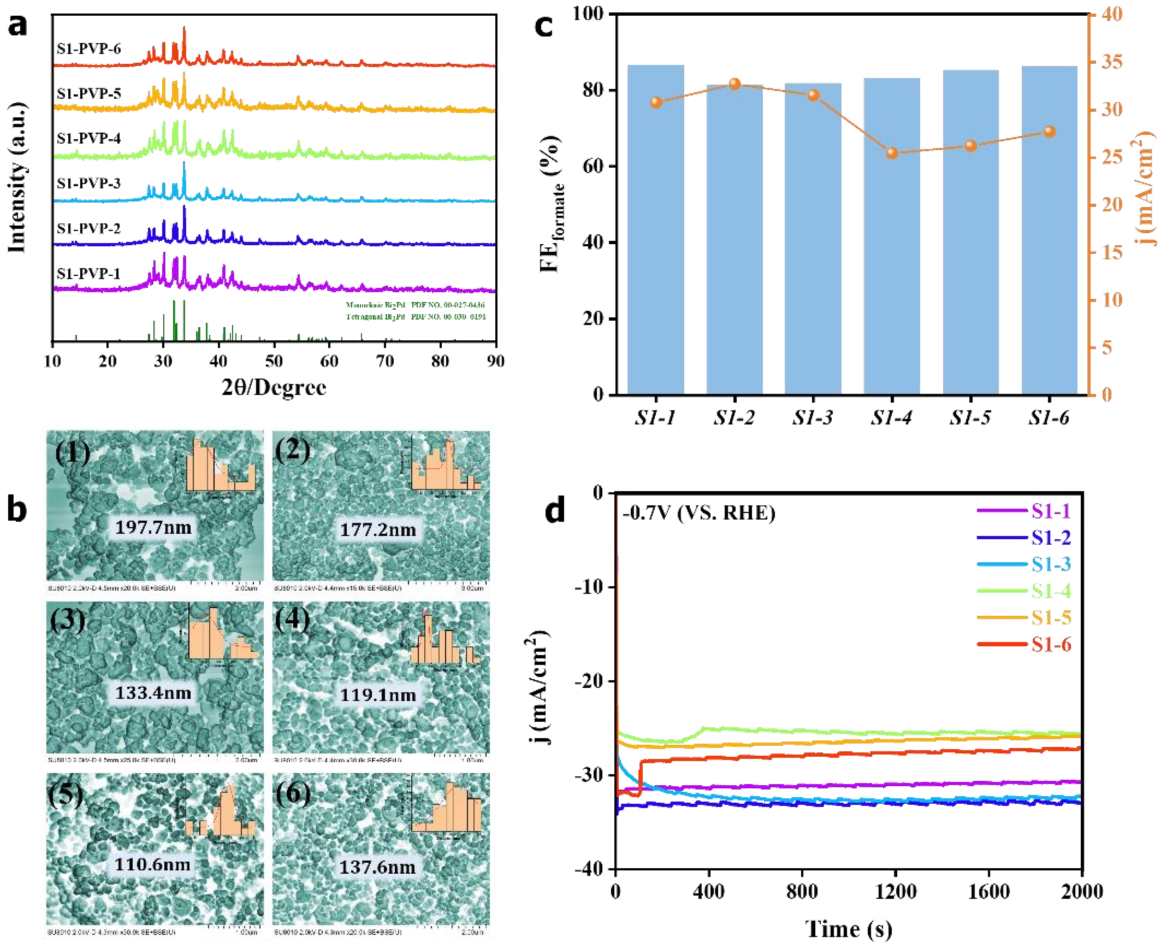

Supplementary Fig.20| The exploration of size effect. a XRD patterns **b** SEM images **c** FE and current density at -0.7V (vs. RHE) **d** constant potential (-0.7V vs. RHE) electrolysis of Bi_2Pd with different sizes.

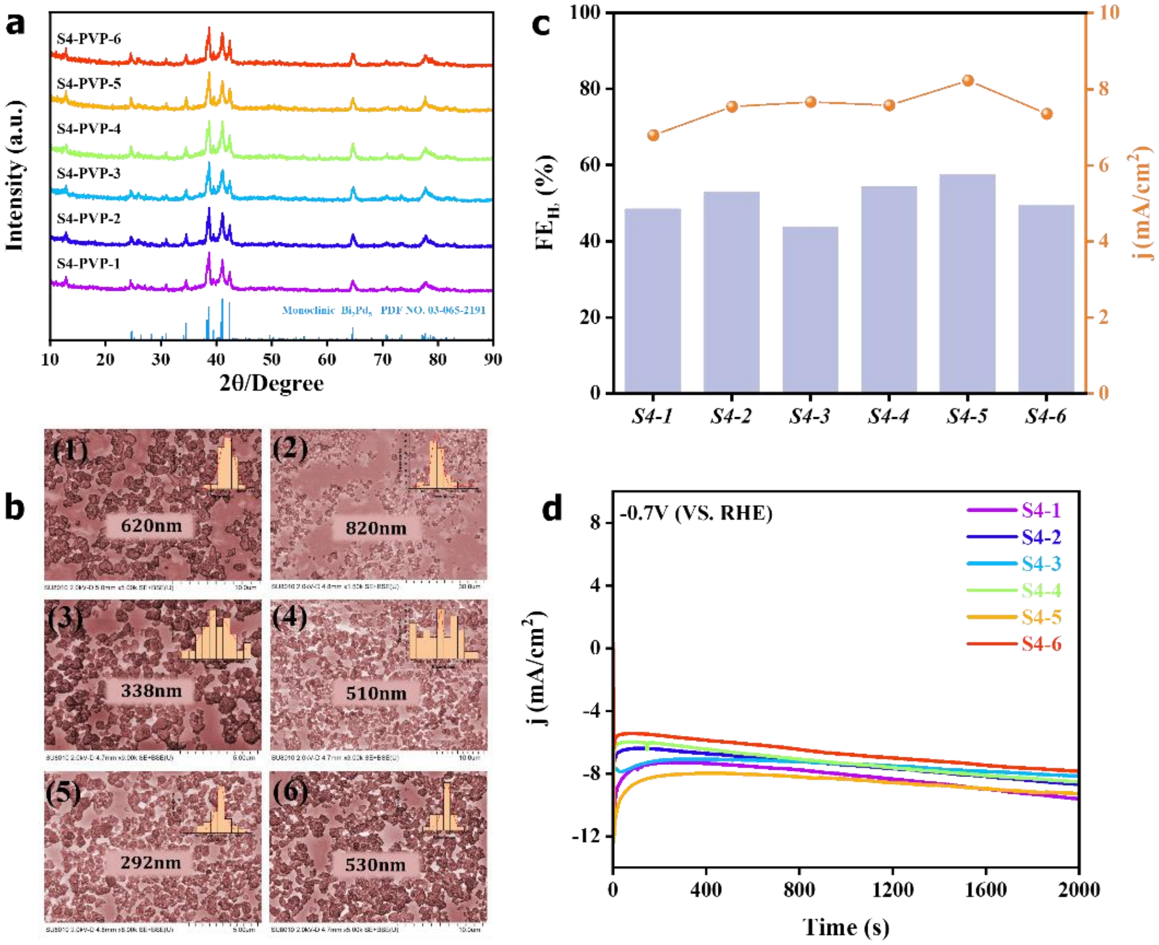

Supplementary Fig.21| The exploration of size effect. a XRD patterns **b** SEM images **c** FE and current density at -0.7V (vs. RHE) **d** constant potential (-0.7V vs. RHE) electrolysis of Bi_2Pd_5 with different sizes.

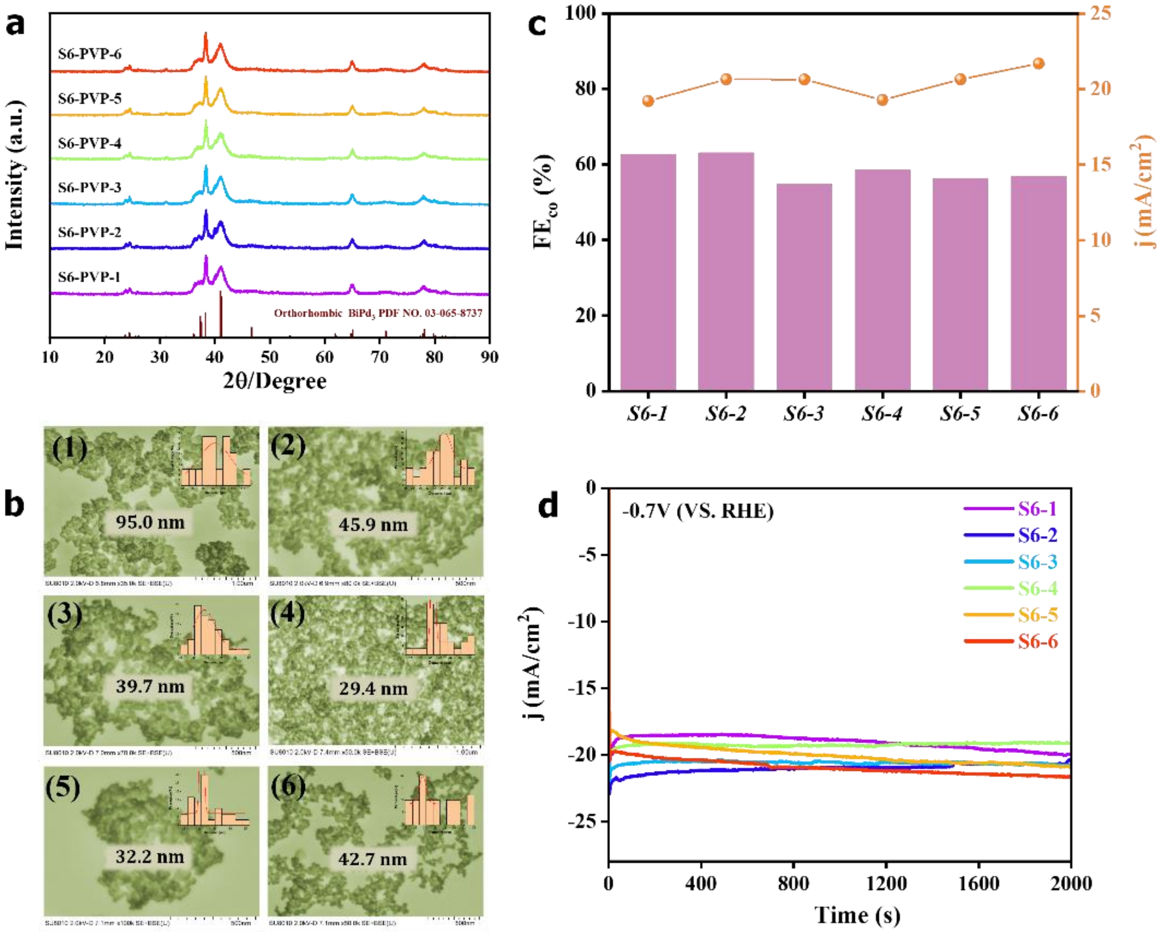

Supplementary Fig.22| The exploration of size effect. a XRD patterns **b** SEM images **c** FE and current density at -0.7V (vs. RHE) **d** constant potential (-0.7V vs. RHE) electrolysis of $BiPd_3$ with different sizes.

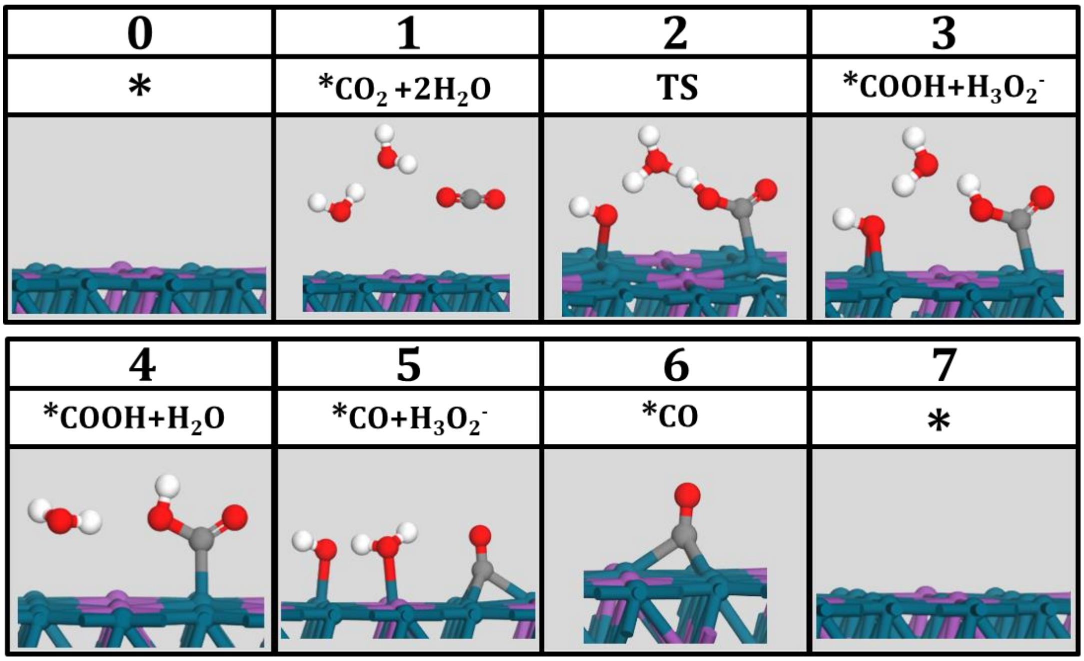

Supplementary Fig.23| DFT calculations. Schematic illustration of the reaction paths for the generation of CO on the (100) face of S6-$BiPd_3$.

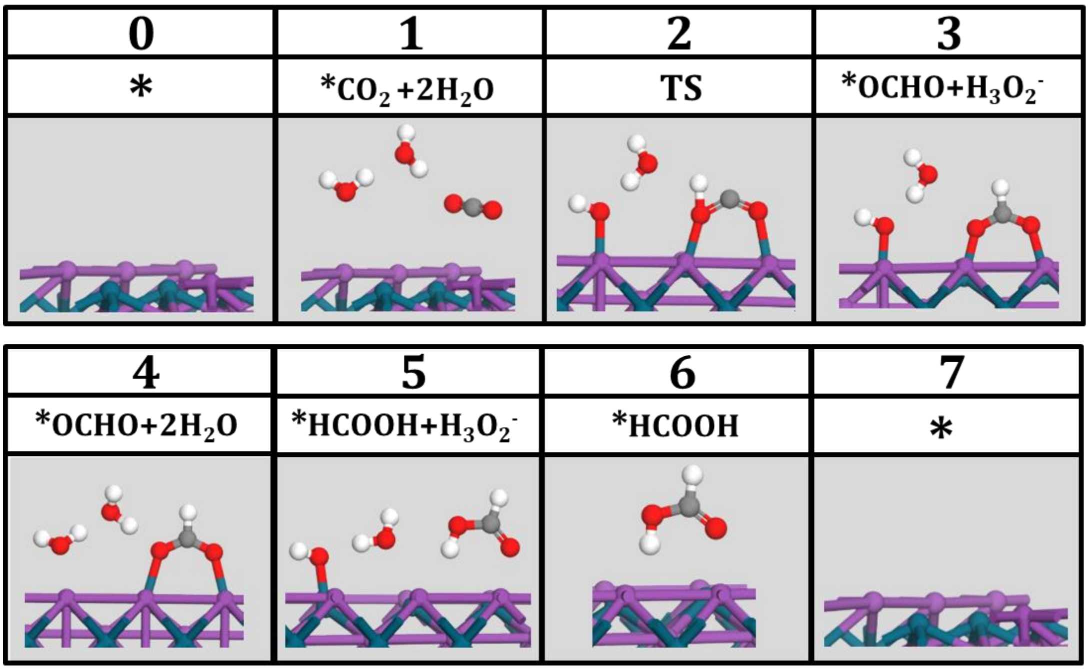

Supplementary Fig.24| DFT calculations. Schematic illustration of the reaction paths for the generation of HCOOH on the (100) face of S1-Bi_2Pd.

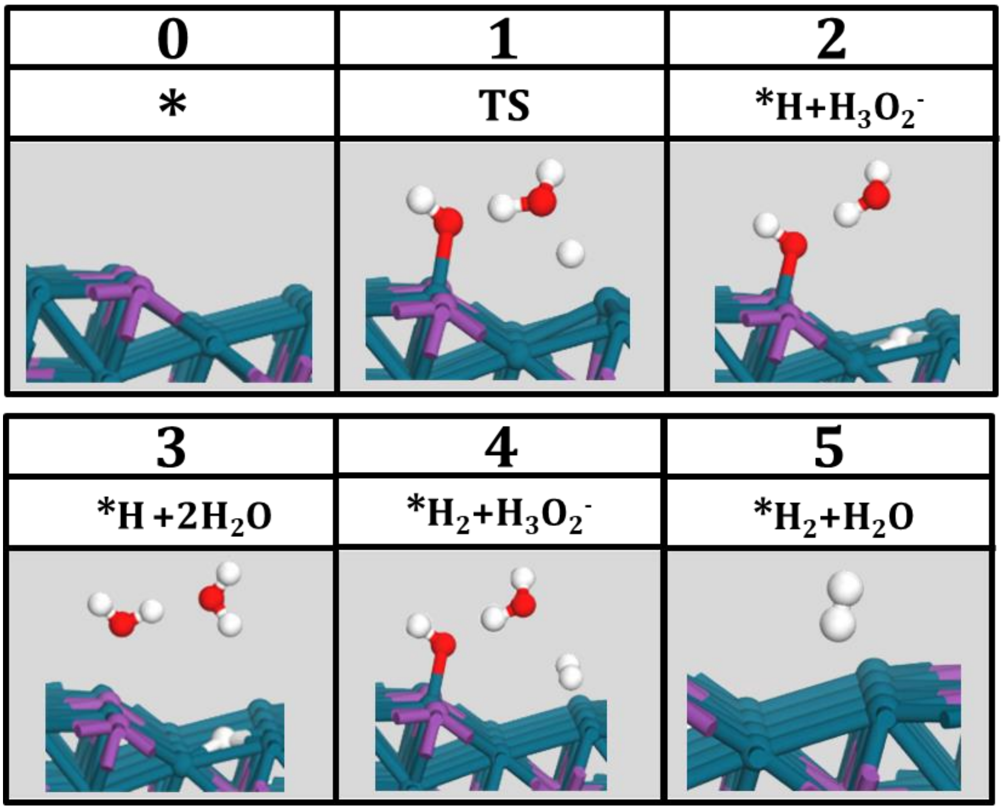

Supplementary Fig.25| DFT calculations. Schematic illustration of the reaction paths for the generation of H_2 on the (001) face of S4-Bi_2Pd_5.

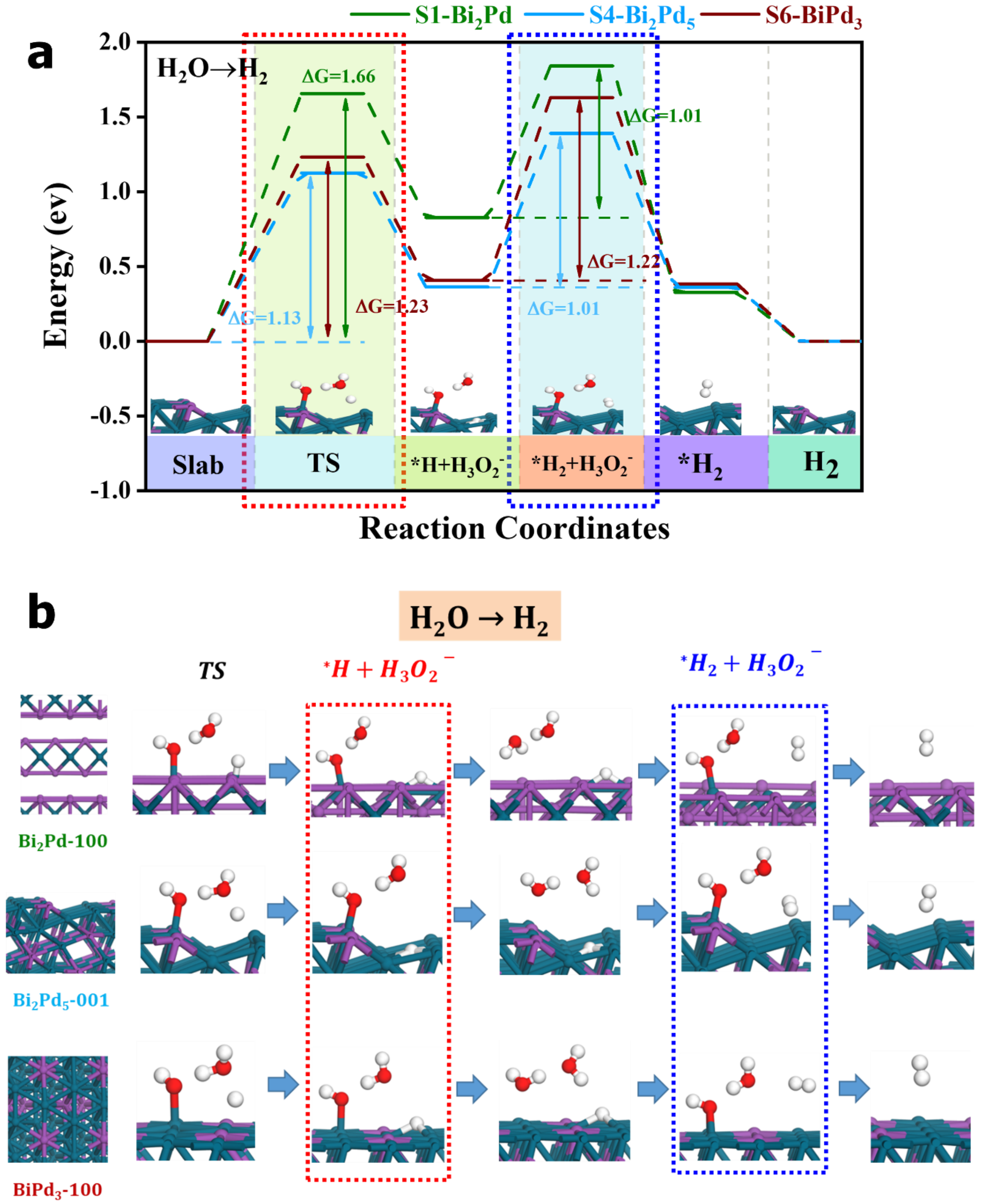

Supplementary Fig.26| DFT calculations. a The calculated free energy diagram of HER on the S1(100) surface, S4(001) surface and S6(100) surface. **b** The optimized atomic structures of reaction intermediates during HER on the S1, S4 and S6.

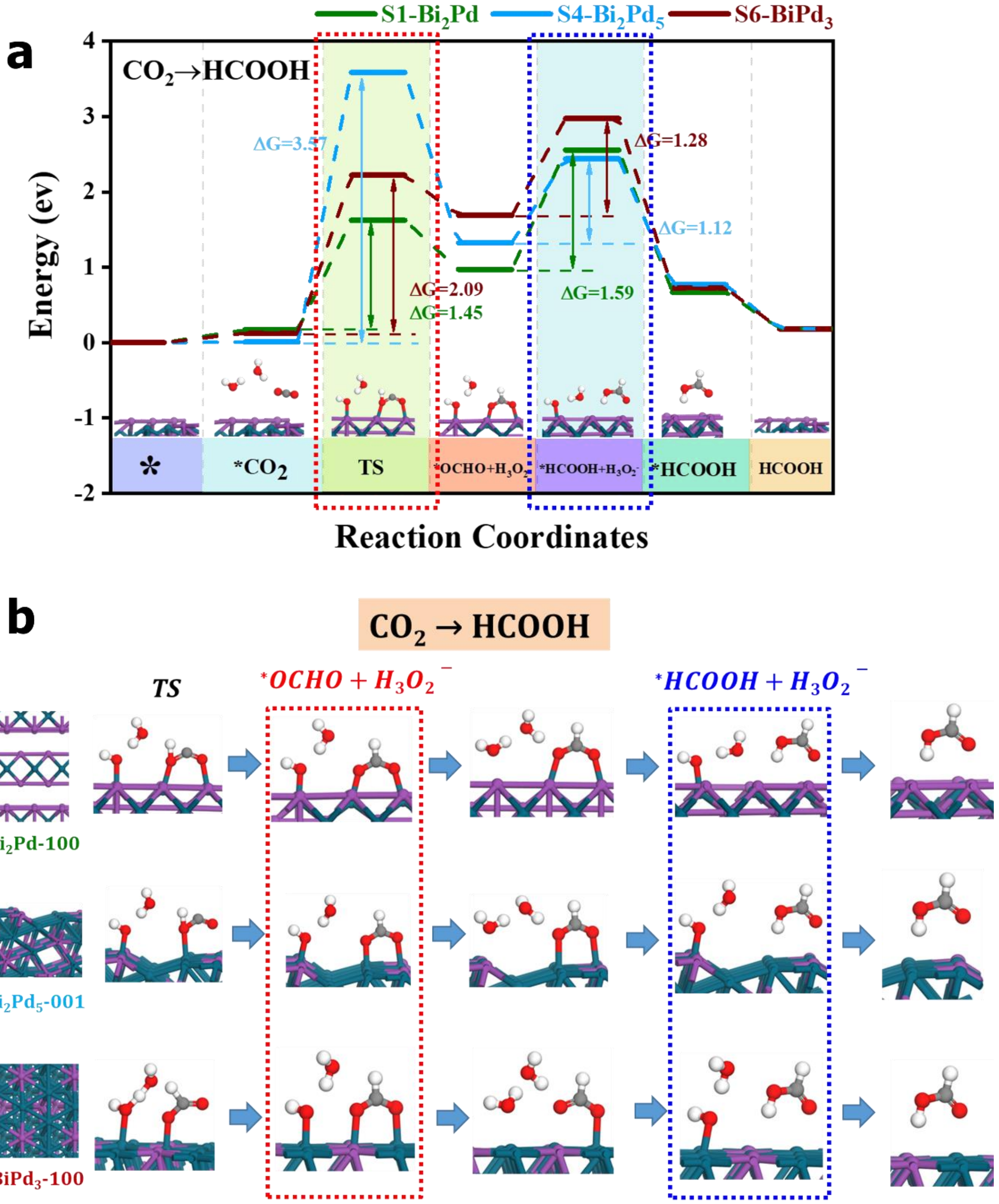

Supplementary Fig.27| DFT calculations. a The calculated free energy diagram of CO_2-to-formate electroreduction on the S1(100) surface, S4(001) surface and S6(100) surface. **b** The optimized atomic structures of reaction intermediates during CO_2-to-formate electroreduction on the S1, S4 and S6.

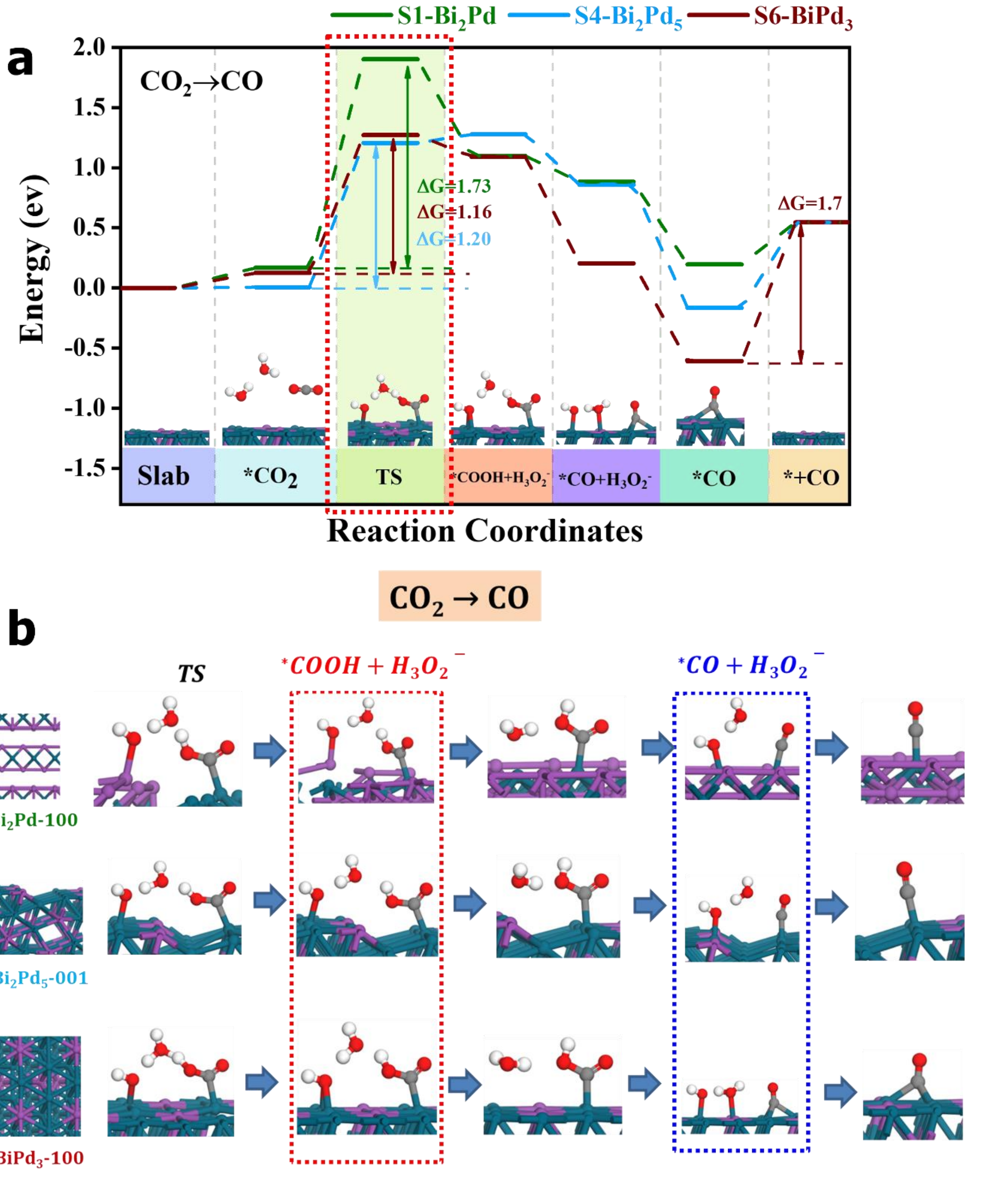

Supplementary Fig.28| DFT calculations. a The calculated free energy diagram of CO_2-to-CO electroreduction on the S1(100) surface, S4(001) surface and S6(100) surface. **b** The optimized atomic structures of reaction intermediates during CO_2-to-CO electroreduction on the S1, S4 and S6.

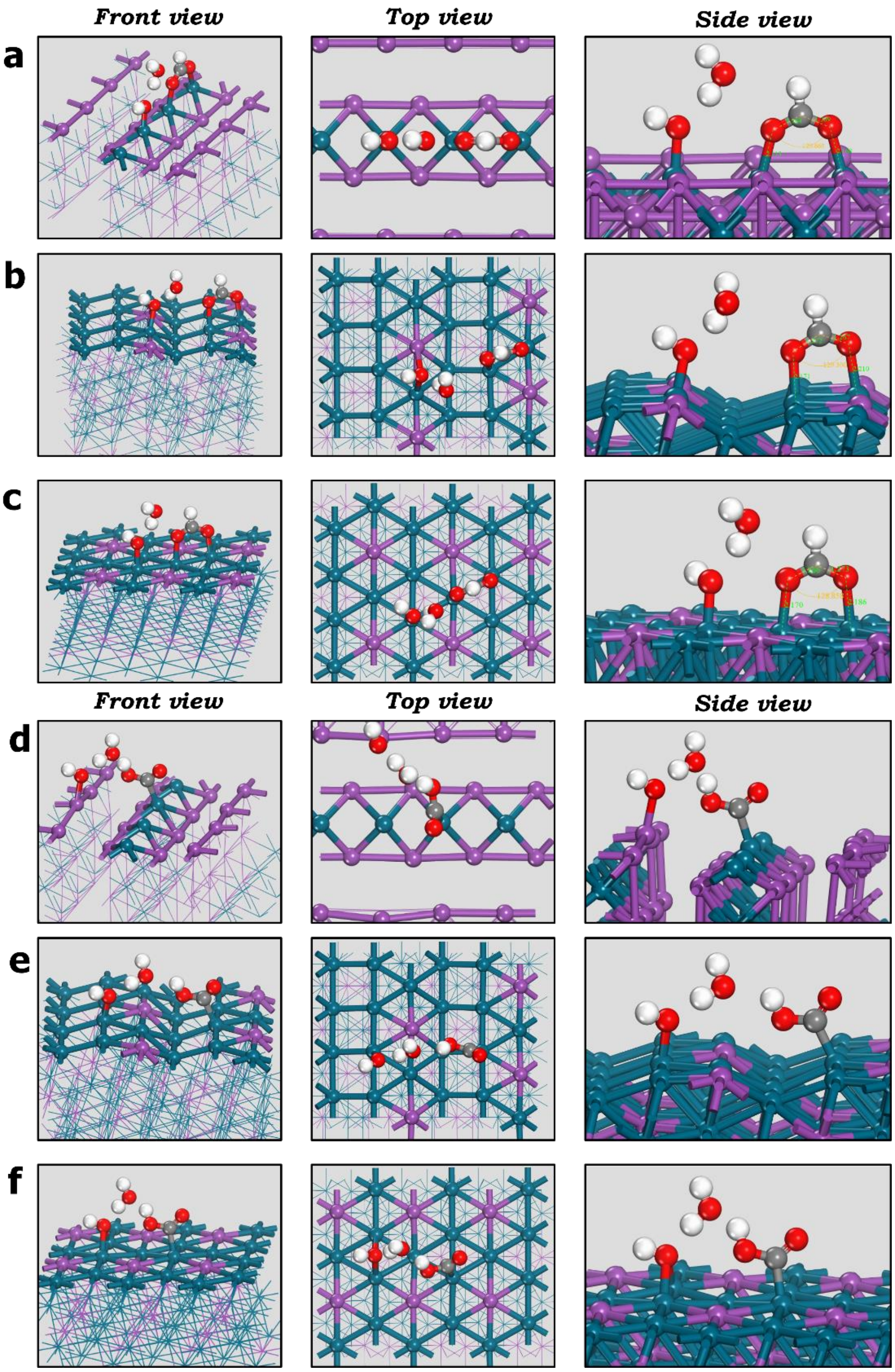

Supplementary Fig.29| DFT calculations. a-c The optimized atomic structure models of adsorbed *OCHO and **d-f** adsorbed *COOH showed along different directions on S1(100), S4(001) and S6(100).

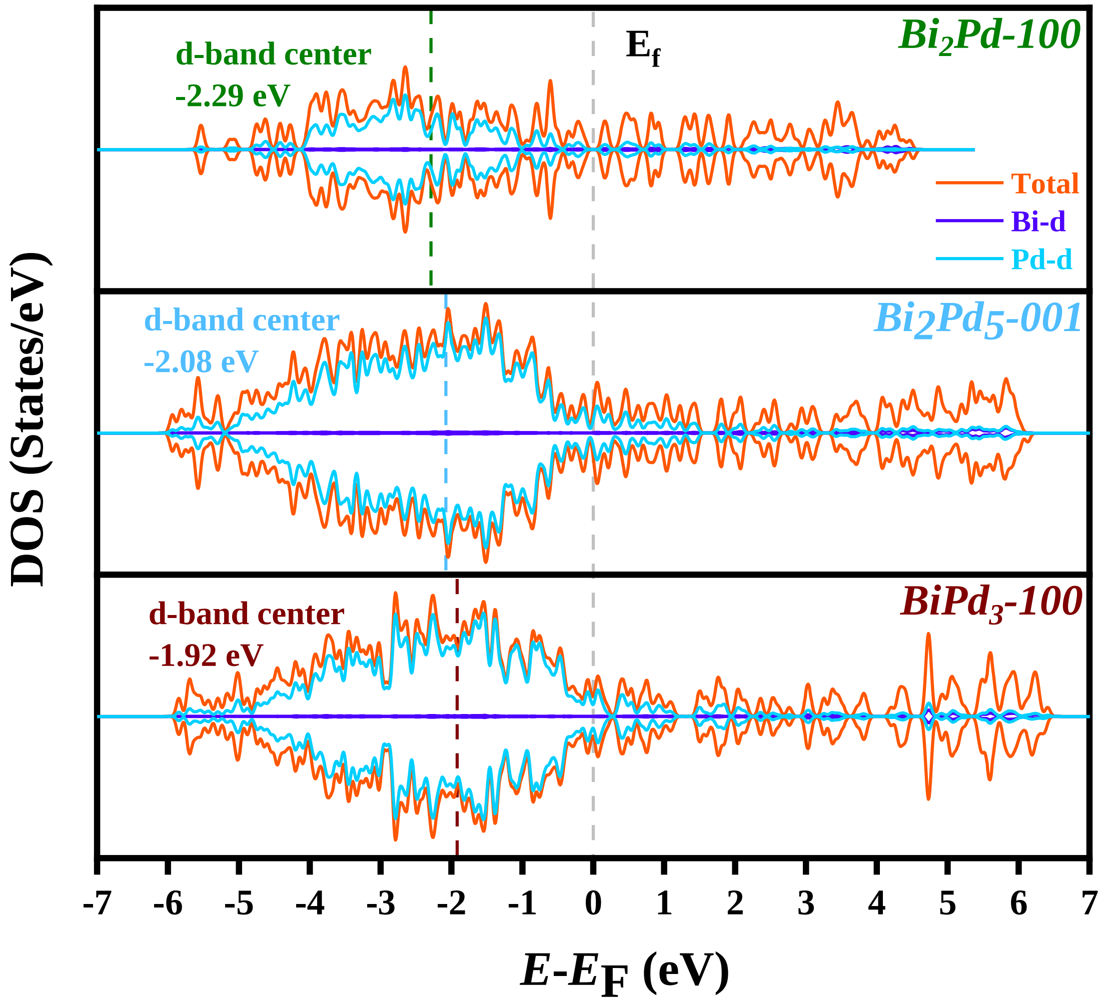

Supplementary Fig.30| DFT calculations. Calculated PDOS of S1, S4 and S6, where the corresponding d-band centers (εd) are labeled with dash lines, and the Fermi level is set to be zero.

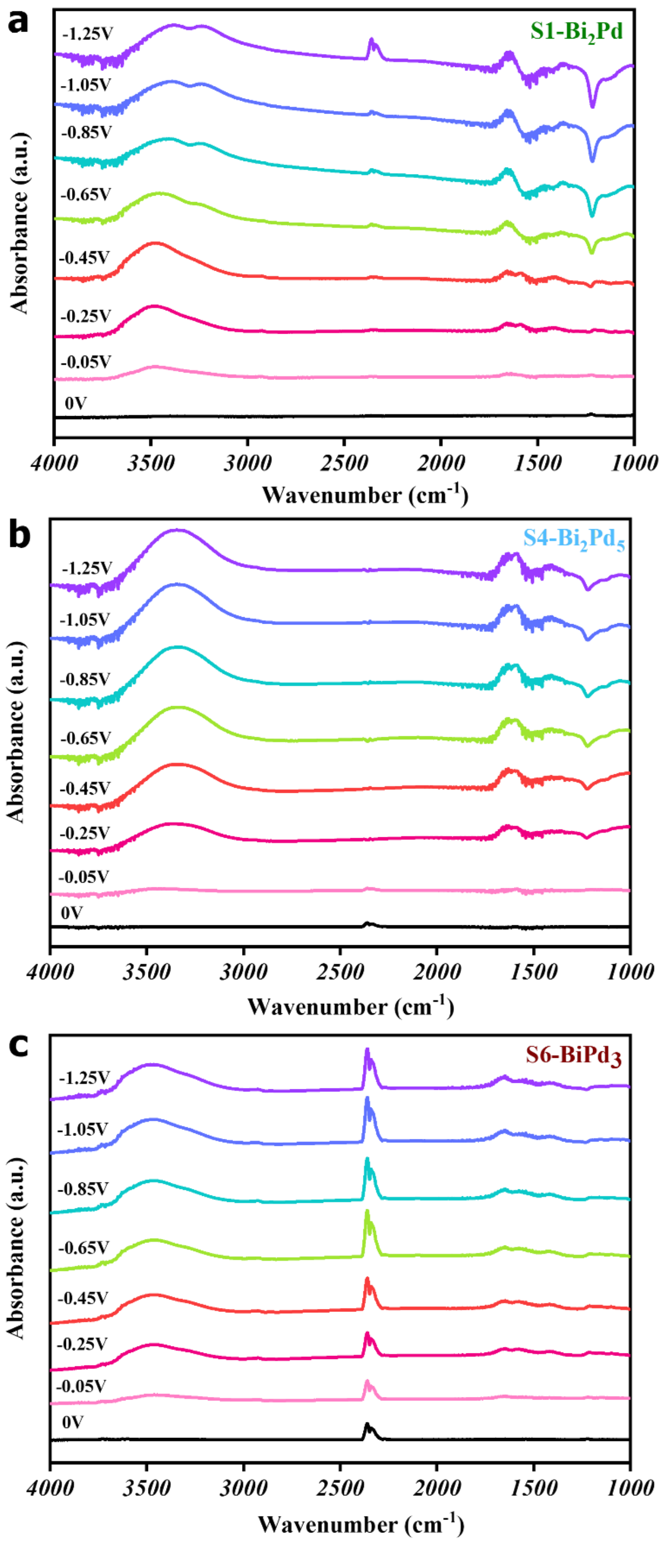

Supplementary Fig.31| Potential-resolved in-situ FT-IR full-spectra of Bi-Pd IMCs. a Bi_2Pd (S1) **b** Bi_2Pd_5 (S4) **c** $BiPd_3$ (S6).

Supplementary Table 1| EXAFS fitting parameters at the Bi L3-edge.

Sample	Shell	N[a]	R(Å)[b]	σ^2 (Å^2)[c]	ΔE_0(eV)[d]	R factor
Bi-Foil	Bi-Bi1	3	3.038	-0.0020	4.634	0.0858
	Bi-Bi2	2	3.267	-0.0027	-9.062	
S1-Bi_2Pd	Bi-Pd1(β)	4	2.632	0.0040	-56.301	0.0049
	Bi-Pd2(α)	1	3.266	0.0059	5.942	
	Bi-Pd3(α)	2	2.526	0.0042	-38.156	
S2-BiPd	Bi-Pd	6	2.569	0.0055	-32.049	0.0980
	Bi-Bi	6	2.814	0.0016	-107.956	
S3-Bi_3Pd_5	Bi-Pd	6	2.196	0.0406	-62.611	0.0651
	Bi-Bi	6	3.267	0.0063	-107.793	
S4-Bi_2Pd_5	Bi-Pd1	1	3.183	-0.0096	-171.381	0.0423
	Bi-Pd2	5	2.686	-0.0074	-45.339	
	Bi-Pd3	4	1.863	-0.0359	-211.793	
S5-Bi_3Pd_8	Bi-Pd1	1	2.186	0.0476	-78.474	0.0486
	Bi-Pd2	9	2.632	0.0112	-19.245	
	Bi-Pd3	3	3.619	0.0196	-9.487	
S6-$BiPd_3$	Bi-Pd	12	2.965	-0.0001	22.991	0.0771
	Bi-Bi	4	3.453	0.0045	-62.232	

Supplementary Table 2| EXAFS fitting parameters at the Pd K-edge.

Sample	Shell	N[a]	R(Å)[b]	σ^2 (Å^2)[c]	ΔE_0(eV)[d]	R factor
Pd-Foil	Pd-Pd1	12	2.741	0.0057	4.187	0.0048
	Pd-Pd2	6	3.848	0.0149	4.462	
	Pd-Pd3	24	4.783	0.0065	7.000	
S1-Bi_2Pd	Bi-Pd	4	2.885	0.0140	-3.814	0.0349
	Bi-Bi	4	3.321	0.0391	-157.198	
S2-BiPd	Bi-Pd	6	2.868	0.0154	-2.490	0.0469
	Bi-Bi	6	3.370	0.0276	-58.283	
S3-Bi_3Pd_5	Bi-Pd	6	2.832	0.0153	-7.147	0.0261
	Bi-Bi	6	3.197	-0.0086	-310.628	
S4-Bi_2Pd_5	Bi-Pd1	1	2.482	0.0052	-24.679	0.0073
	Bi-Pd2	5	2.810	0.0117	-5.734	
S5-Bi_3Pd_8	Bi-Pd1	1	2.452	0.0088	-28.415	0.0114
	Bi-Pd2	9	2.798	0.0115	-5.734	
S6-$BiPd_3$	Bi-Pd	12	2.797	0.0103	-5.948	0.0251

Supplementary Table 3| The reaction paths of the transformation of CO_2-to-CO.

$* + CO_2 + 2H^+ + 2e^- \rightarrow * + CO + H_2O$	
$* + CO_2 + 2H_2O \rightarrow {}^*CO_2 + 2H_2O$	(1)
${}^*CO_2 + 2H_2O + e^- \rightarrow TS$	(2)
$TS \rightarrow {}^*COOH + H_3O_2^-$	(3)
${}^*COOH + H_3O_2^- + H^+ \rightarrow {}^*COOH + 2H_2O$	(4)
${}^*COOH + H_2O + e^- \rightarrow {}^*CO + H_3O_2^-$	(5)
${}^*CO + H_3O_2^- + H^+ \rightarrow {}^*CO + 2H_2O$	(6)
${}^*CO \rightarrow * + CO$	(7)

Supplementary Table 4| The reaction paths of the transformation of CO_2-to-HCOOH.

$* + CO_2 + 2H^+ + 2e^- \rightarrow * + HCOOH$	
$* + CO_2 + 2H_2O \rightarrow {}^*CO_2 + 2H_2O$	(1)
${}^*CO_2 + 2H_2O + e^- \rightarrow TS$	(2)
$TS \rightarrow {}^*OCHO + H_3O_2^-$	(3)
${}^*OCHO + H_3O_2^- + H^+ \rightarrow {}^*OCHO + 2H_2O$	(4)
${}^*OCHO + 2H_2O + e^- \rightarrow {}^*HCOOH + H_3O_2^-$	(5)
${}^*HCOOH + H_3O_2^- + H^+ \rightarrow {}^*HCOOH + 2H_2O$	(6)
${}^*HCOOH \rightarrow * + HCOOH$	(7)

Supplementary Table 5| The reaction paths of the HER.

$* + 2H^+ + 2e^- \rightarrow * + H_2$	
$* + 2H_2O + e^- \rightarrow TS$	(1)
$TS \rightarrow {}^*H + H_3O_2^-$	(2)
${}^*H + H_3O_2^- + H^+ \rightarrow {}^*H + 2H_2O$	(3)
${}^*H + 2H_2O + e^- \rightarrow {}^*H_2 + H_3O_2^-$	(4)
${}^*H_2 + H_3O_2^- + H^+ \rightarrow {}^*H_2 + 2H_2O$	(5)
${}^*H_2 \rightarrow * + H_2$	(6)
